# Maternal postnatal depressive symptoms and offspring emotional and behavioural development at age 7 years in a UK-birth cohort: the role of paternal involvement

**DOI:** 10.1037/dev0001482

**Published:** 2022-11-17

**Authors:** I. Culpin, G. L. Hammerton, A. Stein, M. H. Bornstein, H. Tiemeier, T. Cadman, E. Fredriksen, J. Evans, T. Miller, E. Dermott, J. E. Heron, H. M. Sallis, R. M. Pearson

**Keywords:** ALSPAC, maternal postnatal depression, paternal postnatal depression, paternal involvement, offspring development, population-based study

## Abstract

There is considerable variability in developmental outcomes of children whose mothers experience depression. Few longitudinal studies have examined contributions of paternal involvement in the association between maternal postnatal depression (PND) and offspring development. We examined pathways from maternal PND at 8 weeks (Edinburgh Postnatal Depression Scale; total score) to offspring emotional and behavioural development at 7 years (Strengths and Difficulties Questionnaire; total score) through behavioural, affective and cognitive dimensions of paternal involvement in a UK-based birth cohort (Avon Longitudinal Study of Parents and Children; n=3,434). Analyses were adjusted for baseline confounders and paternal postnatal depression (PND; Edinburgh Postnatal Depression Scale; total score) as an intermediate confounder. Maternal PND was strongly associated with offspring development, but this association was not mediated by the combination of all indirect pathways through various dimensions of paternal involvement. Only father-child conflict emerged as a risk factor for adverse offspring development and as a mediator in the association between maternal PND and offspring development (albeit the effect size was small). If found causal, interventions that reduce father-child conflict may reduce the risk of adverse development in offspring of mothers with PND.

## Maternal Postnatal Depression, Offspring Development and Mechanisms of Transmission

A large body of research has now documented the high prevalence of maternal postnatal depression (PND; Ertel et al., 2011; Horowitz & Goodman, 2004), the link to adverse offspring emotional, behavioural and cognitive development across the lifespan (Dachew et al., 2021; Neri et al, 2020), and associated deleterious health and societal consequences (Bauer et al., 2016). The rapid emotional and cognitive changes that children undergo at this stage of development may make them particularly vulnerable to the adverse effects of maternal PND (Bagner et al., 2020; Goodman & Garber, 2017).

However, outcomes of children whose mothers experience PND are not consistently poor, and effect sizes for the associations are mostly moderate or small (Stein et al., 2014). Insights into factors that accentuate or ameliorate the risks may improve our understanding of intergenerational transmission of mental health difficulties in families and contribute to the development of targeted interventions to reduce adverse impact of maternal PND on offspring development. Parenting is a key modifiable environmental mechanism (Kaminski et al., 2008) that explains some of the adverse effects of maternal PND on the child, representing an intervention target of particular importance (Goodman & Garber, 2017). Evidence suggests that maternal PND disrupts maternal sensitivity and involvement (Murray et al., 2010), which, in turn, are associated with offspring emotional and behavioural problems (Giallo et al., 2014). The importance of paternal mental health (Ramchandani et al., 2005) and involvement (Diniz et al., 2021) for offspring development in the context of ‘normative’ family functioning is increasingly recognised. However, longitudinal studies that examine the role of paternal involvement in the association between maternal PND and offspring development (Goodman et al., 2014) are lacking. Paternal involvement may be of particular importance for mothers and infants in families with mothers experiencing PND through support of the mother and care of the infant (Goodman et al., 2014).

## Paternal Involvement and Offspring Development in the Context of Maternal Postnatal Depression

Both ‘compensatory/buffering’ and ‘spillover’ hypotheses have been articulated to describe possible roles of fathers in the context of maternal PND (Goodman et al., 2014). In line with a family-level resilience perspective (Patterson, 2002), fathers may try to compensate for maternal PND by becoming more involved, ‘buffering’ the adverse effects of maternal PND on the child (Mezulis et al., 2004). Conversely, maternal PND may have a ‘spillover’ effect by negatively affecting paternal involvement and increasing the risk of adverse offspring outcomes (Goodman et al., 2014). Evidence for ‘compensatory/buffering’ effects of paternal involvement on offspring outcomes is inconsistent. Some studies report an increase in positive, warm and sensitive father-child interactions in families where mothers experience PND compared to families where mothers are not affected by PND (Edhborg et al., 2003; Vakrat et al., 2018), but other studies report no evidence that paternal involvement is protective (i.e., moderates the association between maternal PND and offspring development; Carro et al., 1993; Chabrol et al., 1996). The ‘spillover’ hypothesis has also been substantiated with some evidence linking maternal PND to lower levels of paternal involvement with their infants (Paulson et al., 2011).

Family Systems Theory, where the family is viewed as systems of relationships with each individual/subsystem in the family influencing and being influenced by the others (White et al., 2018), is the theoretical framework that underlies both hypotheses. Arguably, with the birth of the child and emergence of substantial postnatal caregiving needs, interdependency in the parental couple system increases, with maternal PND influencing paternal involvement, which, in turn, may influence the relationship between maternal PND and offspring development. Furthermore, mothers who experience PND engage in caregiving tasks to a greater extent than fathers, who are more likely to withdraw from caregiving and reduce involvement when experiencing PND. Thus, it is not always the case that fathers assume a compensatory/buffering role when mothers are affected by depression (Paulson et al., 2006). It is important to provide more insights into the characteristics of paternal involvement when mothers experience PND. However, evidence regarding the impact of maternal PND on the nature and quality of paternal involvement is scant, and, to the best of our knowledge, no large population-based studies to-date have examined a possible mediating role of paternal involvement for child development in the context of maternal PND. Lack of research attention on paternal involvement as a potential mechanism in this context is an important omission given growing evidence emphasising the importance of paternal involvement in numerous aspects of offspring development (Lamb, 2010) and persistent calls for including fathers in scientific and clinical approaches to family functioning affected by maternal PND (Masten & Monn, 2015).

## Paternal Involvement as a Multidimensional Construct

Paternal involvement is multidimensional, as conceptual definitions long ago departed from traditional breadwinning roles to ‘intimate’ fatherhood that prioritises emotional closeness and the quality of the father-child relationship (Dermott, 2014). Paternal involvement is also no longer solely defined as time spent together (Parke & Cookston, 2019), with more nuanced facets, including direct nurturant caregiving and disciplining, as well as emotional and practical support of the mother, being linked to improved offspring emotional and behavioural outcomes (Lamb, 2010). New conceptualisations of paternal involvement include child-focused behavioural (e.g., direct involvement in caregiving), affective (e.g., quality of the father-child relationship, such as enjoyment, warmth and conflict), and cognitive (e.g., parenting confidence and beliefs regarding caregiving) dimensions (Marsiglio et al., 2000; Pleck, 2010). Aspects of paternal involvement that are shaped by maternal influences and mother-father relationship (e.g., ‘gatekeeping’, a complex phenomenon defined as maternal beliefs and practices that discourage or facilitate paternal involvement; Miller, 2018) have also been described. Supporting the mother through sharing household tasks and responsibilities is another important dimension of paternal involvement, with significant consequences for the child (Parke & Cookston, 2019). Similar to mothers, fathers are also under pressure to balance caregiving with demands of paid work, childcare and family life (Lamb, 2013; Miller, 2010) often accompanied by mothers’ perceptions that fathers are not ‘pulling their fair share’ with this benchmarking influencing paternal involvement (Pleck, 2012).

Recognition that child-focused and mother-influenced paternal involvement are important for offspring development represents another marked shift in fatherhood scholarship (Lamb, 2010), explicitly acknowledging that the father-child relationship is embedded in a network of relationships among fathers, mothers and children (Cabrera et al., 2014). However, few studies have examined paternal involvement as a multidimensional construct that captures cognitive, affective and behavioural aspects as well as maternal influences (Schoppe-Sullivan et al., 2004). Failures to do so preclude establishing the individual relative contributions of various dimensions of paternal involvement to offspring development (Lamb, 2010). We address this limitation by examining several dimensions of child-focused and mother-influenced paternal involvement in relation to offspring emotional and behavioural outcomes in the context of maternal PND.

## Importance of Paternal Postnatal Depression

**Figure 1:**
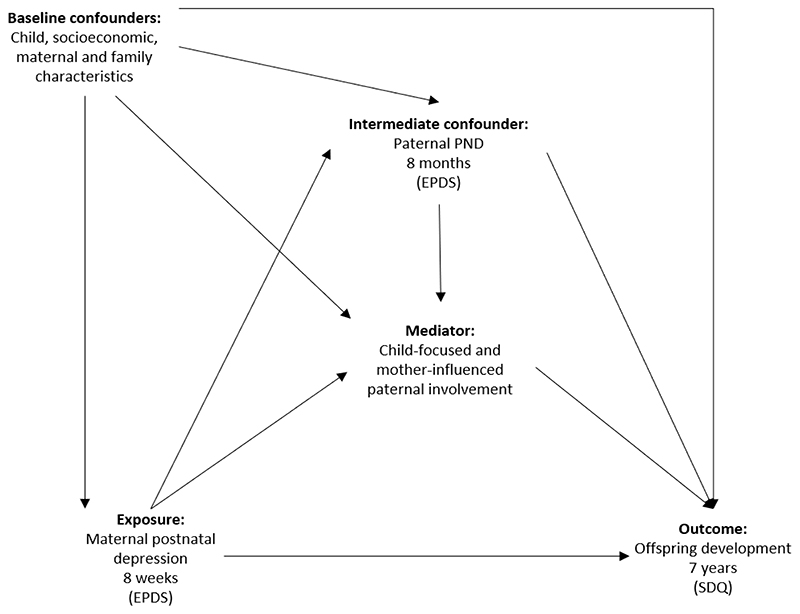
Conceptual diagram for exposure (maternal PND), mediator (child-focused and mother-influenced dimensions of paternal involvement), outcome (offspring development), baseline confounders (child, socioeconomic, maternal and family characteristics) and intermediate confounder^*^ (paternal PND) ^*^ Intermediate confounder: variable induced by the exposure, affecting both the mediator and the outcome, thus confounding mediator-outcome association

Parenting is transactional and dynamic (Bornstein, 2015). Existing research acknowledges reciprocal influences and the interplay between paternal involvement and the child, with offspring response to parenting shaped by temperamental and biological dispositions, which may evoke variations in parenting behaviours (Avinun & Knafo, 2014). In addition, paternal PND and its impact on offspring development in the context of maternal PND is often overlooked. Maternal depression in late pregnancy and during the postnatal period are associated with increased risk of paternal PND (Fredriksen et al., 2019), which in turn influence paternal parenting (Wilson & Durbin, 2010) and offspring emotional and behavioural development (Ramchandani et al., 2005), thus potentially influencing the association between paternal involvement (mediator) and offspring development (outcome); Figure 1 presents conceptual pathways between maternal and paternal PND, paternal involvement and offspring development. Paternal and maternal PND may increase offspring risk of adverse development (Goodman et al., 2014), while absence of paternal PND may be a protective factor reducing adverse effects of maternal PND on paternal involvement and offspring outcomes. Mezulis et al. (2004) found that paternal PND exacerbated adverse effects of maternal PND on offspring behavioural problems, but this moderating effect was limited to those fathers affected by depression who spent considerable time caring for their children. To the best of our knowledge, no longitudinal study has examined the role of paternal PND in the associations between maternal PND, paternal involvement and offspring development. We focused on the first year of the child’s life, which may be a sensitive period of increased vulnerability to the adverse effects of maternal PND and parenting on offspring development (Bagner et al., 2010). This is a time when most infants are highly dependent on parental care and involvement. In order to satisfy temporal precedence assumptions that underly mediational models, the earliest time point of maternal PND was chosen to align with the earliest assessment of paternal involvement items (8 weeks), while paternal PND was assessed at 8 months and offspring development at 7 years.

## The Current Study

The current study aims to build and expand on existing research by examining the role of paternal involvement as a potential mediating mechanism that could transmit the adverse effects of maternal PND on offspring development. We took advantage of rich longitudinal data on paternal parenting collected in a large UK-based birth cohort study, the Avon Longitudinal Study of Parents and Children (ALSPAC) to (1) model child-focused and mother-influenced dimensions of paternal engagement and (2) estimate the extent to which the association between maternal PND at 8 weeks and offspring emotional and behavioural difficulties at 7 years is mediated by child-focused and mother-influenced dimensions of paternal involvement during the first four years of the child’s life. Quantifying this association has important implications for the design and development of preventative and intervention programmes, given the modifiable nature of parenting (Kaminski et al., 2008). The unique nature of the rich longitudinal ALSPAC data enabled us also to account for a range of confounding factors, including child polygenic score for neuroticism (PGS) as a proxy for genetic confounding that may indicate possible child evocative effects, and paternal PND as a possible intermediate confounder. It is important to note that the study focused on parents with postnatal depressive symptoms (as assessed by the Edinburgh Postnatal Depression Scale; Cox et al., 1987), and not a clinical diagnosis of depression.

## Method

### Study Cohort

**Figure 2:**
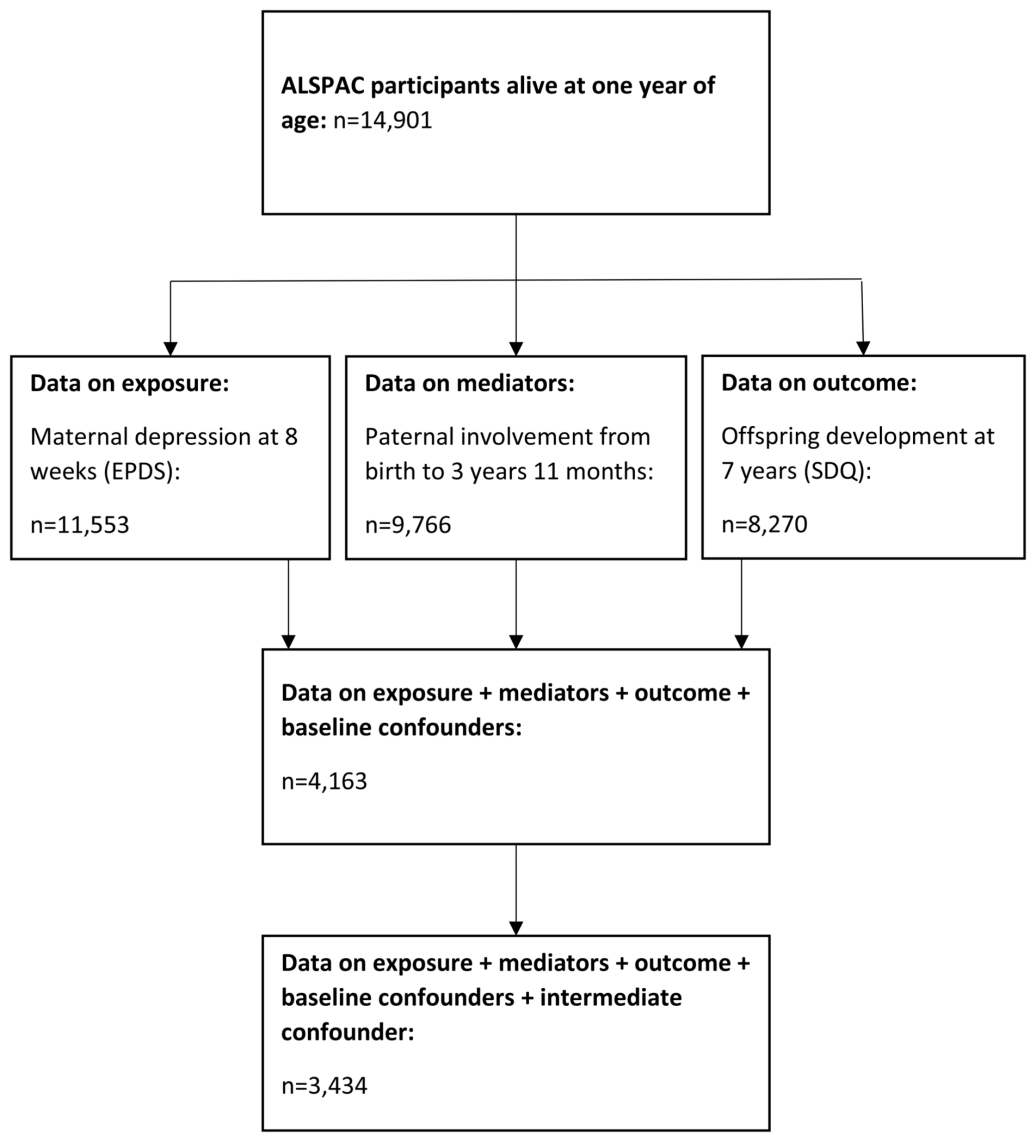
Flow chart depicting study sample derivation

The sample comprised participants from the ALSPAC cohort. During Phase I enrolment, 14,541 pregnant mothers residing in the former Avon Health Authority in the south-west of England with expected dates of delivery between 1 April 1991 and 31 December 1992 were recruited. The total sample size is 15,454 pregnancies, of which 14,901 were alive at 1 year of age. Our study sample comprised 3,434 participants with at least one parenting item and complete data on exposure, outcome and confounding factors (Figure 2). Ethical approval and informed consent for the data collection were obtained from the ALSPAC Ethics and Law Committee and the Local Research Ethics Committees (http://www.bristol.ac.uk/alspac/researchers/research-ethics/). Information about ALSPAC is available at www.bristol.ac.uk/alspac/, including a fully searchable data dictionary (http://www.bris.ac.uk/alspac/researchers/our-data/). Further details on the cohort profile, representativeness and phases of recruitment are described in three cohort-profile papers (Boyd et al., 2013; Fraser et al., 2013; Northstone et al., 2019).

### Measures

#### Exposure: Maternal Postnatal Depression

In line with previous research (Neri et al., 2020; Wilcox et al., 2021), we refer to maternal PND not as a clinical diagnosis, but as experiences of self-reported depressive symptoms measured at 8 weeks postnatally using the Edinburgh Postnatal Depression Scale (EPDS; Cox et al., 1987), a 10-item self-reported depression questionnaire validated for use during the perinatal period. To make full use of variation in symptoms, individual depression items were summed to derive a continuous score (range 0-30). The continuous score was used in all analyses, except descriptive characteristics of the study sample by exposure status (threshold >13; 10.1%; Table 1). Further details on maternal depressive symptoms assessed using the EPDS in the ALSPAC cohort, including stability across assessment time points and predictive validity, are provided in the previously published data note (Paul & Pearson, 2020).

#### Outcome: Offspring Emotional and Behavioural Development

Offspring emotional and behavioural development were assessed using the Strengths and Difficulties Questionnaire (Goodman, 1997) completed by the mothers of the study children at age7 years). The scale consists of 25 questions with five subscales (prosocial [i.e., engagement in positive social interactions], emotional symptoms, conduct problems, hyperactivity and peer problems), which have been extensively validated, demonstrating high consistency, reliability and diagnostic predictability (Goodman, 2001). Consistent with previous research, the first 4 subscales were combined to derive a total difficulties score used in all analyses (Gutierrez-Galve et al., 2015).

#### Mediators: Child-Focused and Mother-Influenced Dimensions of Paternal Involvement

**Figure 3:**
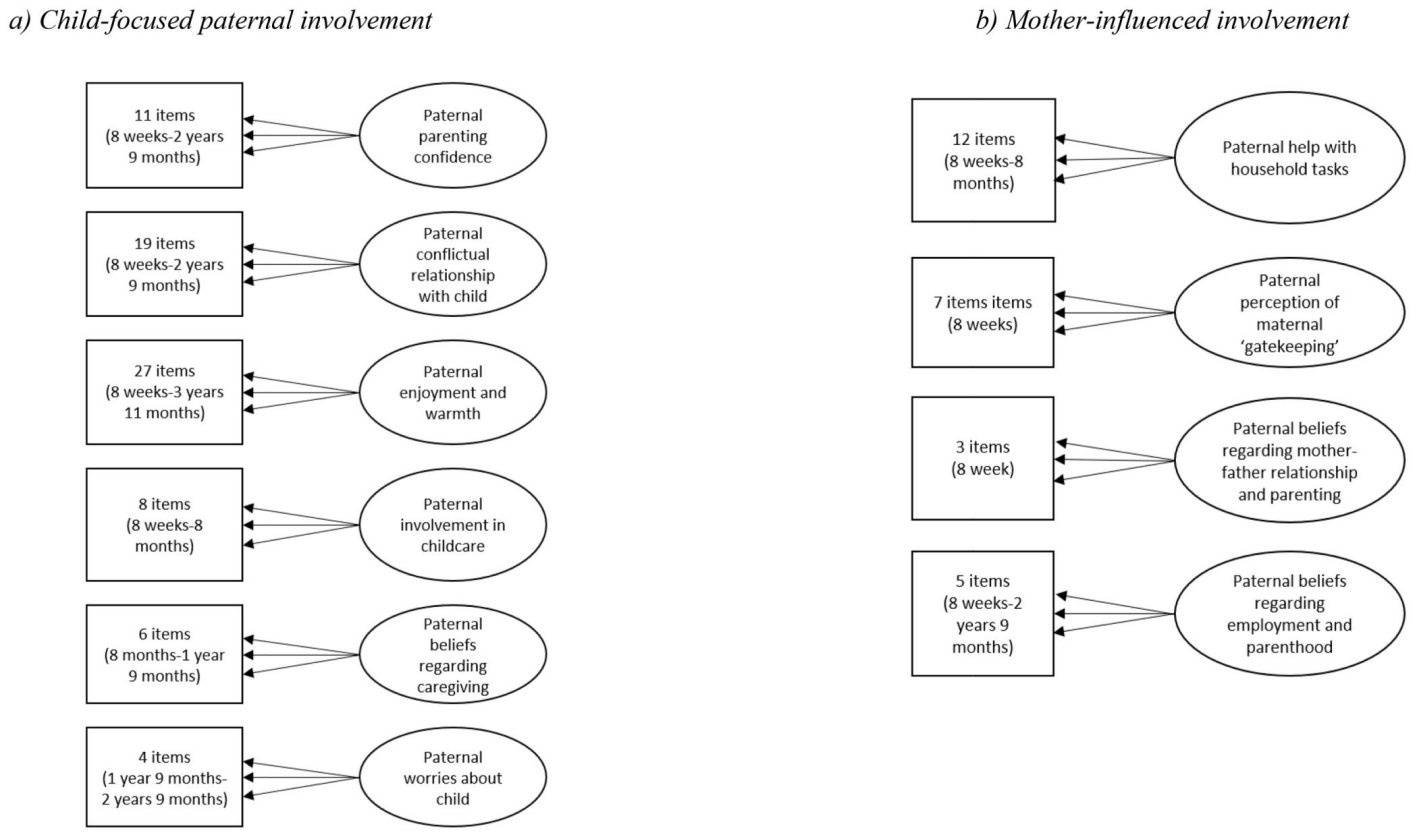
Hypothesised latent factor (CFA) measurement model representing specific factors capturing child-focused and mother-influenced dimensions of paternal involvement

**Figure 4:**
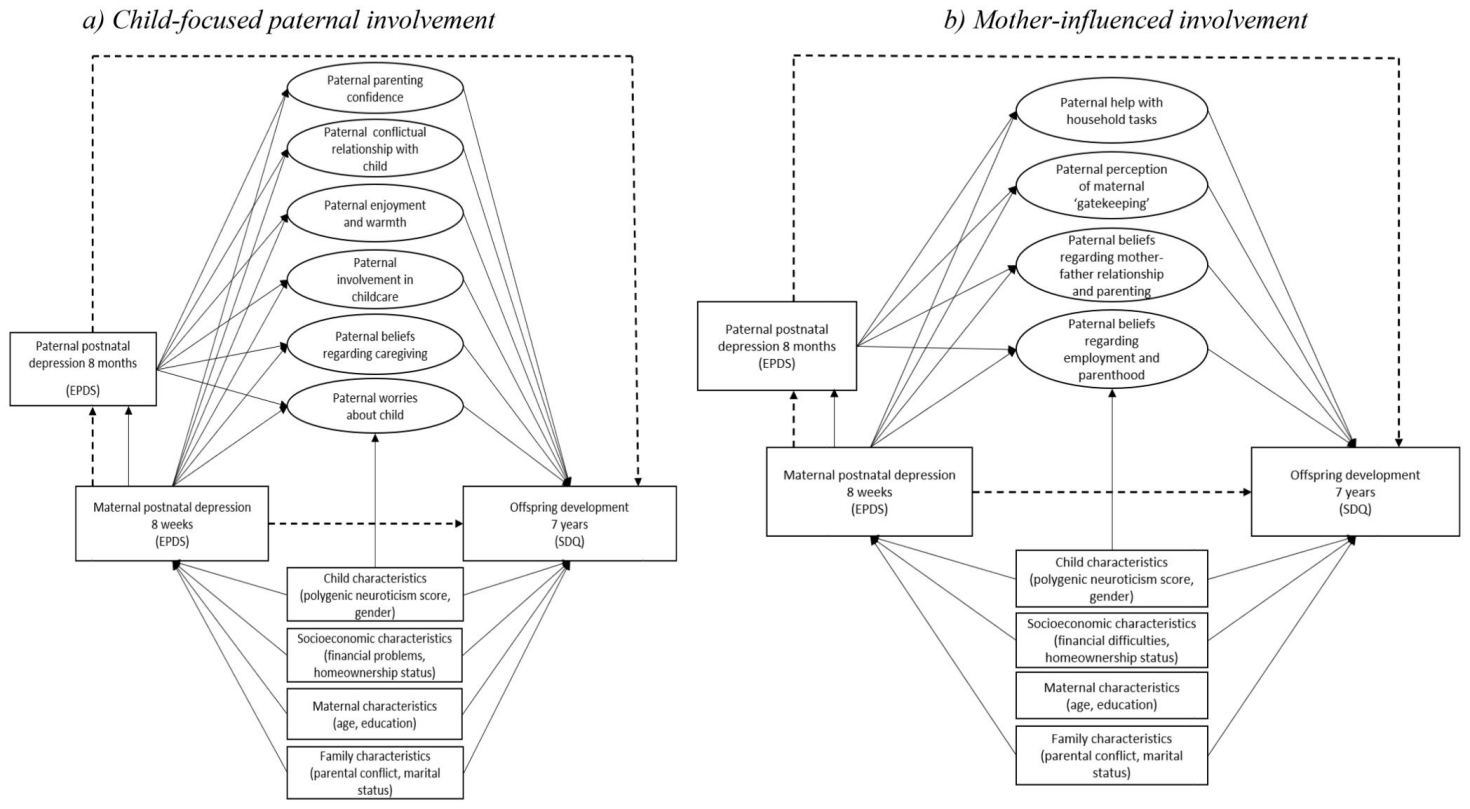
Structural equation mediation model estimating the direct effect of maternal postnatal depression on offspring development at age 7 years and the indirect effect through specific factors capturing child-focused and maternal-influenced dimensions of paternal involvement, adjusted for child PRS, gender, antenatal baseline confounders, and paternal PND as an intermediate confounder *Note:* Observed variables are represented by squares. Latent variables are represented by circles. Error terms covariances, correlations between the factors and individual items loading onto each specific factor are not represented to reduce figure complexity. Pathways that constitute total indirect effect are represented by bold lines; pathways that constitute direct effect are represented by dashed lines.

Full details pertaining to item selection and theoretical development of factors are presented in Methods S1. In summary, potential parenting items (>150) were extracted from paternal self-reported questionnaires administered on 5 occasions from birth to age 3 years 11 months. Conceptual factor underpinnings were drawn from extensive empirical and sociological literature on paternal engagement in infancy (Dermott, 2014; Marsiglio et al., 2000; Palkovitz, 2019; Pleck, 2012). In line with a revised conceptualisation of paternal involvement (Pleck, 2010; Schoppe-Sullivan et al., 2004), individual items fell into two theoretically distinct sources of paternal involvement: (1) *child-focused* paternal involvement capturing behavioural (e.g., direct involvement in caregiving), affective (e.g., quality of father-child relationship, such as enjoyment, warmth and conflict, worries about the child) and cognitive (e.g., parenting confidence and beliefs regarding caregiving) dimensions directed at the child; and (2) *mother-influenced* paternal involvement with the child through the lens of maternal expectations (e.g., maternal ‘gatekeeping’, managing employment and parenthood), mother-father relationship (e.g., paternal beliefs regarding mother-father relationship and its impact on parenting) and indirect material care through support of the mother (e.g., paternal help with household tasks and responsibilities). The hypothesised latent factor (CFA) models capturing child-focused (6 dimensions), and mother-influenced (4 dimensions) paternal involvement are represented in Figure 3a-b, with derived factors, items, standardised factor loadings and fit indices for the measurement models presented in Tables S1-S2. Two separate measurement models including CFA-based factors (Figure 3a-b) and structural mediation models including pathways between constructs (Figure 4a-b) were fitted to capture potentially differential mediating roles of child-focused and mother-influenced paternal involvement in the association between maternal PND and offspring development.

#### Potential Baseline Confounders: Child Polygenic Score for Neuroticism, Socioeconomic, Parental and Family Characteristics

Analyses were adjusted for child polygenic score (PGS) for neuroticism to account for possible child genetic correlations including evocative associations with parenting (Mills-Koonce et al., 2007). However, it should be noted that polygenic scores capture only a small fraction of heritability, thus they do not comprehensively account for possible genetic confounding (Pingault et al., 2021). Genotyped data were available for 8,237 children in the ALSPAC cohort (full details in Methods S1). Disadvantaged socioeconomic status, marital status and conflict are also strong risk factors for maternal PND (Milgrom et al., 2008), less optimal paternal parenting practices (Leigh & Milgrom, 2008), and adverse offspring emotional and behavioural development (Stein et al., 2014). In consequence, we adjusted for a range of antenatal prospectively measured potential confounding factors extracted from maternal questionnaires, including: highest maternal educational attainment (minimal education or none/compulsory secondary level [up to age 16 years; O-Level], non-compulsory secondary level [up to age 18 years; A-Level/university level education]), maternal age in years, presence of financial difficulties or no financial difficulties, homeownership status (owned/mortgaged, private/council [subsidised public housing] rented), marital status (married, never married) and a continuous score capturing parental conflict (higher scores representing higher levels of conflict). Given the primary exposure of interest was maternal PND and lower response rates for fathers compared to mothers, as well as moderate to strong correlations between maternal and paternal education (r=0.6, p≤0.001) and age (r=0.7, p≤0.001), analyses were adjusted for mother-reported baseline confounders only to avoid introducing missing data.

#### Potential Intermediate Confounder: Paternal Postnatal Depression

We accounted for possible baseline antenatal confounders of the exposure-outcome, exposure-mediator and mediator-outcome associations by including them in the regression models to estimate each of these pathways (Northstone et al., 2019). However, evidence suggests that maternal depression in pregnancy and during the postnatal period is associated with increased risk of paternal PND, which in turn influences paternal parenting, and offspring emotional and behavioural development, acting as a potential exposure induced intermediate confounder of the mediator-outcome association (Sheikh et al., 2016). Similarly to maternal PND, we refer to paternal PND not as a clinical diagnosis, but as self-reported experiences of depressive symptoms during the postnatal period. Failure to account for intermediate confounders may result in biased inferences regarding direct and indirect (mediated) effects (De Stavola et al., 2015; Loeys et al., 2014). Thus, we accounted for paternal PND assessed using the Edinburgh Postnatal Depression Scale (EPDS; Cox et al., 1987) as a continuous score at 8 months postnatally (threshold ≥12; 7.3% (Ramchandani & Psychogiou, 2009); full details in Methods S1). Further details on assessment of paternal PND using the EPDS in the ALSPAC cohort, including stability across assessment points, predictive validity and correlations between maternal and paternal PND, are provided in the previously published data note (Paul & Pearson, 2020).

### Statistical Analysis

#### Latent Factor Models

Full details of latent factor models describing derivation of factors capturing child-focused and mother-influenced paternal involvement are presented in Methods S1. In summary, individual parenting items that were primarily theoretically relevant and had standardised loadings >0.15 were loaded onto the hypothesised parenting dimensions and modelled using Confirmatory Factor Analyses (CFA), a subset of SEM with a robust Weighted Least Square (WLSMV) estimator in M*plus* recommended to model both categorical and continuous data (Muthén & Asparouhov, 2013). The Root Mean Square Error of Approximation (RMSEA; >0.06) and Comparative Fit Index and Tucker-Lewis Index (CFI/TLI; >0.95) were used to evaluate the fit of the models (Hu & Bentler, 1998). The chi-square test of overall fit is prone to model misspecification when sample size is large (Schumacker & Lomax, 2004); thus, we gave preference to relative fit indices.

#### Direct and Mediated Effects

Full details of the mediation model, including path-specific direct and indirect effects are described in Methods S1. In summary, we examined the extent to which the association between maternal PND (8 weeks) and offspring emotional and behavioural development (7 years) is mediated by child-focused (six latent factors; Figure 4a) and mother-influenced (four latent factors; Figure 4b) dimensions of paternal involvement using Structural Equation Modelling (SEM) in M*plus* v.8.3 (Muthén & Muthén, 2015). Analyses of the mediation models were restricted to participants with complete data on exposure (maternal PND), outcome (offspring emotional and behavioural development), child PGS, baseline (socioeconomic, maternal and family characteristics) and intermediate (paternal PND) confounders (*n*=3,434).

First, we estimated the unadjusted models composed of exposure, outcome and mediators only. Second, we estimated models adjusted for child PGS and all antenatal baseline confounders (Model 1), and further adjusted for paternal PND (8 months) as a possible intermediate confounder of the mediator-outcome association (Model 2). Path-specific effects of interest representing pathways that constitute total indirect (bold lines) and direct (dashed lines) effects are described in Figure 4a-b and Methods S1. Indirect effects [95% CIs] were calculated using the product-of-coefficients method and bias-corrected (BC) bootstrapping (*n*=1,000 replications) to account for the non-normal distribution (MacKinnon et al., 2004). Results from path analyses with continuous score (offspring total difficulties score), including indirect effects, are presented as unstandardised regression coefficients (hereafter referred to as *β*). Both results from Model 1 (adjusted for child PRS and antenatal baseline confounders) and Model 2 (further adjusted for paternal PND at 8 months as an intermediate confounder) are presented for comparison of estimates. However, preference is given to Model 2 under the assumption that Model 1 is biased without the inclusion of paternal PND.

#### Missing Data: Multiple Imputation

We conducted sensitivity analyses to examine the impact of missing data due to loss to follow-up on our findings. Full description of imputation methods, including characteristics of the sample by the completeness of data, are presented in Methods S1 and Table S4.

## Results

### Sample Characteristics

Characteristics of the study sample and offspring total difficulties mean score at age 7 years by exposure status (maternal PND at 8 weeks) are presented in Table 1. Mothers who reported higher levels of education (A-Level/University Degree) were more likely to experience PND than mothers with lower levels of educational attainment. However, younger, never married mothers, those who reported higher levels of inter-parental conflict and financial difficulties, and those residing in private and/or council rented accommodations were more likely to report PND. Offspring of those mothers who experienced PND had higher mean total difficulties scores.

### Paternal Involvement Factors

CFA models capturing child-focused and mother-influenced dimensions of paternal involvement showed an adequate model fit (RMSEA: child-focused involvement: 0.022, 95%CI 0.021 to 0.022; mother-influenced involvement: 0.044, 95%CI 0.043 to 0.045; CFI/TLI: child-focused involvement: 0.904/0.899; mother-influenced involvement: 0.957/0.950; Tables S1-S2) supporting further tests of structural paths (direct and mediated effects). Six factors capturing child-focused and four factors capturing mother-influenced dimensions of paternal involvement were derived (full details in Results S1), with associations between child-focused and mother-influenced factors presented in Table S3).

#### Child-Focused Paternal Involvement

*Factor 1 Paternal parenting confidence:* 11 items relating to paternal feelings of confidence in the parenting role and perceptions of the ability to engage effectively in parenting behaviours, with higher factor scores representing higher levels of paternal parenting confidence.

*Factor 2 Paternal conflictual relationship with child:* 19 items relating to conflict, harsh disciplining, irritation with the child and feelings of being overwhelmed, with higher factor scores signifying lower levels of conflictual parent-child relationship, irritation with the child and less harshness in paternal disciplining.

*Factor 3 Paternal enjoyment and warmth:* 27 items relating to feelings of enjoyment, affection, love and warmth toward the child, with higher factor scores representing more paternal enjoyment, affection and warmth toward the child.

*Factor 4 Paternal involvement in childcare:* 8 items describing frequency of paternal involvement in various aspects of childcare, with higher factor scores representing higher frequency of paternal involvement in childcare.

*Factor 5 Paternal worries about child:* 4 items pertaining to paternal worries about the child, with higher factor scores representing lower levels of paternal worries about the child.

*Factor 6 Paternal beliefs regarding caregiving:* 6 items relating to paternal beliefs regarding caregiving, including endorsement of structure (e.g., regularity and routines in infant care) and attunement (e.g., responsiveness to infant cues), with higher factor scores representing more paternal appreciation of structure and higher levels of attunement to child’s cues.

#### Mother-Focused Paternal Involvement

*Factor 1 Paternal help with household tasks:* 12 items related to various aspects of paternal help with household tasks, with higher scores representing higher levels of paternal help with household tasks since the child was born.

*Factor 2 Paternal perception of maternal ‘gatekeeping’:* 7 items relating to paternal perceptions of maternal beliefs and behaviours that encourage or hinder paternal involvement in childcare, with higher factor scores indicating higher levels of paternal perceptions of being supported and included in childcare by the mother (i.e., less maternal ‘gatekeeping’).

*Factor 3 Paternal beliefs regarding mother-father relationship and parenting:* 3 items relating to paternal beliefs and perceptions regarding the impact that the birth of the child has had on the father-mother relationship, with higher factor scores representing paternal beliefs more concordant with positive changes in the nature of the mother-father relationship.

*Factor 4 Paternal beliefs regarding employment and parenthood:* 5 items relating to paternal beliefs regarding maternal expectations around employment, childcare and paternal difficulties in managing childcare and employment, with higher scores indicating paternal beliefs concordant with less pressure to look after child after work and fewer difficulties with managing childcare and employment.

### Associations Between Maternal PND, Child-Focused and Mother-Influenced Dimensions of Paternal Involvement and Offspring Emotional and Behavioural Development

Our pathways of interest were those between maternal PND and offspring emotional and behavioural development at age 7 years through child-focused and mother-influenced dimensions of paternal involvement. Specifically, we examined the pathways from maternal PND to paternal involvement (both direct and through paternal PND), and the pathways from paternal involvement to offspring emotional and behavioural development (Figure 4a-b represent pathways that constitute direct and total indirect effects) with full description of estimates for Model 1 provided in Results S1.

#### Exposure (Maternal PND) – Mediator (Paternal Involvement) Associations

To summarise, in Model 1 (adjusted for child PGS and antenatal baseline confounders; Table 2) maternal PND at 8 weeks was strongly associated with less paternal parenting confidence, more father-child conflict, less paternal enjoyment and warmth, more paternal worries about the child, higher levels of perceived maternal ‘gatekeeping’, more negative beliefs regarding the impact the birth of the child had on the mother-father relationship, and paternal feelings of more pressure to look after the child after work and more struggles to manage childcare and employment.

In contrast, in Model 2 (further adjusted for paternal PND at 8 months as an intermediate confounder; Table 3), maternal PND was strongly associated with more paternal enjoyment and warmth (β=0.055, 95% CI: 0.031, 0.078, p≤0.001) and direct involvement in childcare (β=0.029, 95% CI: 0.017, 0.041, p≤0.001), as well as more paternal parenting confidence (β=0.029, 95% CI: 0.004, 0.054, p=0.027) that was not explained through paternal PND. Maternal PND was also associated with less father-child conflict (β=0.034, 95% CI: 0.007, 0.061, p=0.016), more paternal help with household tasks (β=0.009, 95% CI: -0.001, 0.019, p=0.070), and more paternal struggle to manage childcare and employment (β=-0.010, 95% CI: -0.019, -0.001, p=0.047), although 95% CIs were wide, that was not explained through paternal PND. Maternal PND was also strongly associated with paternal PND, which, in turn, was strongly associated with less paternal parenting confidence, less enjoyment and warmth, less involvement in childcare and appreciation of regular routine, more father-child conflict and more paternal worries about the child, less paternal help with household tasks, higher levels of perceived maternal ‘gatekeeping’, more negative feelings regarding the impact the birth of the child on the mother-father relationship, paternal feelings of more pressure to look after the child after work and more struggles to manage childcare and employment (Table 3; estimates presented in Results S1).

#### Mediator (Paternal Involvement) – Outcome (Offspring Emotional and Behavioural Development) Associations

In Model 1, lower levels of paternal parenting confidence, warmth and enjoyment, higher levels of father-child conflict and worries about the child, and paternal difficulties to manage childcare and employment were associated with higher risk of offspring emotional and behavioural difficulties at age 7 years (Table 2). In Model 2, however, the only child-focused dimension of paternal involvement, higher levels of father-child conflict, was strongly associated with higher risk of offspring emotional and behavioural difficulties at age 7 years (β=-0.546, 95% CI: -0.998, -0.093, p=0.018; Table 3), with no evidence for associations between any of the mother-influenced dimensions of paternal involvement and offspring emotional and behavioural development.

#### Direct and Mediated Effects

Full description of estimates for direct and mediated effects in Model 1 is presented in Results S1. To summarise, in Model 1 (Table 4), there was evidence of a total indirect effect from maternal PND to offspring emotional and behavioural development at age 7 years through the combination of all child-focused, but not mother-influenced, dimensions of paternal involvement. There was strong evidence of total and direct effects from maternal PND to offspring development in models capturing both child-focused and mother-influenced paternal involvement. There was evidence for specific indirect effects through paternal parenting confidence, paternal conflictual relationship with the child, paternal enjoyment and warmth, paternal worries about the child, and paternal difficulties to manage childcare and employment (although 95% CIs were wide).

In contrast, in Model 2 (Table 4), there was no evidence of total indirect effect from maternal PND to offspring development at 7 years through the combination of all factors capturing child-focused (β=0.082, 95%CIs: -0.028, 0.192, p=0.144) and mother-influenced (β=-0.023, 95%CIs: -0.066, 0.020, p=0.282) dimensions of paternal involvement. Similar to Model 1, there was strong evidence of total and direct effects from maternal PND to offspring development in models capturing both child-focused (total effect: β=0.267, 95%CIs: 0.077, 0.457, p<0.001; direct effect: β=0.185, 95%CIs: 0.110, 0.273, p<0.001) and mother-influenced (total effect: β=0.163, 95%CIs: 0.092, 0.234, p<0.001; direct effect: β=0.186, 95%CIs: 0.139, 0.233, p<0.001) paternal involvement. However, unlike Model 1, there was only some evidence for specific indirect effect through paternal conflictual relationship with the child (β=0.055, 95%CIs: 0.001, 0.110, p=0.046), although the 95% CIs were wide, but not other child-focused dimensions of paternal involvement. There was no evidence for specific indirect effects through any of the mother-influenced dimensions of paternal involvement.

## Discussion

### Main Findings

In this population-based cohort study, we modelled multiple child-focused and mother-influenced dimensions of paternal involvement during early childhood and estimated the extent to which they mediated the association between maternal PND and offspring emotional and behavioural difficulties at age 7 years. One strength of our study is the inclusion of paternal PND as a possible exposure-induced intermediate confounder of the mediator-outcome association (see Figure 1 for explanation of conceptual pathways). It has been extensively argued that failure to account for potential intermediate confounders may bias inferences regarding direct and indirect (mediated) effects (De Stavola et al., 2015).

In models that adjusted for child PGS and baseline confounders only, maternal PND was strongly associated with *lower scores* on most child-focused and mother-influenced dimensions of paternal involvement (reflected in negative associations), which mediated a proportion of the association between maternal PND and offspring emotional and behavioural difficulties. This reflects the ‘spillover’ hypothesis, wherein higher levels of maternal PND are associated with lower father involvement (Goodman et al., 2014). However, accounting for paternal PND *reversed* associations between maternal PND and both child-focused and mother-influenced dimensions of paternal involvement (resulting in direct positive associations between maternal PND and paternal involvement). Specifically, once paternal PND was accounted for, maternal PND was associated with *higher scores* on child-focused and mother-influenced paternal involvement, including higher levels of paternal parenting confidence, enjoyment and warmth toward the child, less father-child conflict, more paternal involvement in childcare and more help with household tasks. The reversal of the associations (from negative to positive) between maternal PND and dimensions of paternal involvement, once paternal PND was accounted for, should be interpreted with caution given the possibility that this is due to a suppression effect (Tu et al., 2008). A suppression effect is when the association between an exposure (e.g., maternal PND) and an outcome (e.g., paternal involvement) changes in direction when a third variable (e.g., paternal PND) is adjusted for. The positive (adjusted) association is the independent effect of maternal PND on paternal involvement, after removing the impact of paternal PND on paternal involvement. Given that our *a priori* causal model (Figure 1) considered paternal PND to be on the causal pathway between maternal PND and paternal involvement, it is the total (negative) association between maternal PND and paternal involvement that we consider to be clinically relevant (and which we used to estimate our indirect effects of maternal PND on child outcomes).

Our findings indicated that maternal PND was still associated with more paternal struggles to manage employment and childcare responsibilities once paternal PND was accounted for, suggesting that these dimensions of paternal involvement are negatively associated with maternal PND irrespective of paternal PND. This finding is consistent with existing sociological studies that emphasise paternal struggles to maintain a ‘work-life balance’ (Miller, 2010), and a fractious relationship between paid employment and family activities, often placed in opposition to each other (Dermott, 2005).

Amongst all dimensions of paternal involvement, only a conflictual father-child relationship emerged as a risk factor for adverse offspring development, as well as a mediator (albeit the effect size was small), in the association between maternal PND and offspring development irrespective of paternal mental health. There was no evidence for the mediating role of any other child-focused and mother-influenced dimensions of paternal involvement. It has previously been argued that conflictual father-child relationship may undermine children’s sense of emotional security increasing their susceptibility for emotional and behavioural problems even in the absence of paternal PND (Palkovitz, 2019). Paternal PND has also been linked to offspring emotional and behavioural difficulties and father-child conflict (Kane & Garber, 2004), which, in turn, mediates the association between paternal PND and adverse offspring development (Kane & Garber, 2009). Our findings suggest that father-child conflict may also be a pathway of risk transmission in the context of maternal PND even after accounting for levels of paternal depression.

Strong evidence emerged for a direct association between maternal PND and risk of offspring adverse emotional and behavioural development in both models examining child-focused and mother-influenced dimensions of paternal involvement. These findings are consistent with previous research suggesting that paternal non-involvement explained a small proportion of the association between maternal PND and offspring development (Gutierrez-Galve et al., 2015). It may be that other pathways of transmission from maternal PND to offspring development, for instance quality of the mother-child relationship and maternal parenting, may be more important in this context (Connell & Goodman, 2002). Consistent with accumulating epidemiological evidence, maternal PND was associated with increased risk of paternal PND (Fredriksen et al., 2019; Paulson & Bazemore, 2010), which, in turn, was strongly associated with increased risk of adverse offspring emotional and behavioural development (Sweeney & MacBeth, 2016). However, the association between maternal and paternal PND should be interpreted with caution and warrants further investigation, possibly through the widely articulated theoretical lens of assortative mating (Mathews & Reus, 2001). In line with previous research, paternal PND was also strongly associated with reduced child-focused and mother-influenced paternal involvement (Wilson & Durbin, 2010).

### Strengths and Limitations

Strengths of this study include the longitudinal design, large community-based sample and availability of rich repeated self-reported measures of paternal parenting that enabled us to model several dimensions of child-focused and mother-influenced dimensions of paternal involvement across early childhood. Furthermore, we were able to examine possible mediating role of these dimensions in the association between maternal PND and offspring emotional and behavioural development in mediation models adjusted for a range of confounders, including paternal PND and child PGS. To the best of our knowledge, no previous studies have examined dimensions of child-focused and mother-influenced involvement as possible transmission pathways in the association between maternal PND and offspring development while accounting for paternal PND.

Paternal involvement is embedded in complex family systems of intertwined relationships and their impact on the nature of parent-child relationship (Marsiglio et al., 2000). Our conceptualisation of paternal involvement was guided by developmental and sociological literature explicitly acknowledging the complexity of paternal roles and the importance of affective and cognitive, not just behavioural (Palkovitz, 2019), dimensions, as well as the negotiated nature of paternal involvement shaped by significant relationships (Kerig, 2019). Although this perspective renders conceptual and statistical examination of paternal involvement more difficult (Cabrera & Tamis-LeMonda, 2013), it answers the call for multidimensional conceptualisation of father involvement (Palkovitz, 2019), enabling a more nuanced understanding of the variety of forms in which paternal involvement occurs and their individual contributions to child development.

Associations among parental mental health, parenting and offspring development are complex and bidirectional (Mills-Koonce et al., 2007). Our findings suggest that maternal PND is associated with paternal PND, which, in turn, is associated with reduced child-focused and mother-influenced dimensions of paternal involvement. However, in line with transactional developmental models (Bornstein, 2009), children with more difficult emotional and behavioural responses may influence maternal and paternal PND, as well as paternal involvement (Sameroff, 2009). Addressing possible bidirectionality (Xerxa et al., 2020) fell outside the scope of the present study. However, we attempted to account for possible evocative effects and possible shared genetic liability for emotional difficulties in parents and offspring by including a child genetic score for neuroticism, which explained a small proportion of variance in these traits. Polygenic scores capture only a small fraction of trait heritability and, thus, of the confounding due to genetic factors unlikely to reflect true genetic effects (Pingault et al., 2021). Shared genetic variance in depression and parenting not captured by the score may still confound associations between parental PND, paternal involvement and offspring development.

Another limitation is the lack of additional assessments of parental depression at later time points in development and non-independence of measurement. Parental depression may recur across development (Wilcox et al., 2021) and estimating its effects on offspring development from one time-point (8 weeks postnatally) is a limitation of our conceptual model. Furthermore, maternal PND and offspring development were both reported by the mothers. Mothers who experience depression may inaccurately report their children’s emotional and behavioural problems (Ordway, 2011), potentially overestimating the direct effect. The complexity of the model precluded examination of offspring outcome as a multi-informant latent factor (i.e., rated by parents, clinicians and teachers; De Los Reyes & Kazdin, 2005) to address this limitation. However, given the strength of the direct association between maternal PND and offspring emotional and behavioural difficulties modelled as the SDQ total problems score, any multi-informant latent factor would unlikely change this finding. Modelling SDQ as total difficulties score is a well-validated approach widely adopted in developmental research, including studies utilising ALSPAC data (Gutierrez-Galve et al., 2015). Notably, paternal involvement measures were father-reported, reducing the possibility of overestimating mediated effects (i.e., the associations between maternal and paternal PND, paternal involvement and offspring development) due to shared variance bias. The importance of independent data in examining mediating pathways between maternal depression and offspring development has been strongly advocated (Appleyard & Carlson, 2005).

Some parenting involvement items were measured before and/or concurrently with the assessment of paternal PND, offering an alternative explanation whereby paternal involvement influenced paternal PND. However, ample evidence supports the adverse effect of paternal PND on paternal parenting and involvement (Wilson & Durbin, 2010), suggesting that the direction of this association is unlikely to flow in the opposite direction. Nevertheless, this alternative needs to be acknowledged when interpreting our findings.

The lack of independently assessed measures of paternal involvement is also a limitation, potentially biasing the estimation of the associations between paternal PND, dimensions of paternal involvement and offspring emotional and behavioural development (Kendler & Baker, 2007). It has been argued that measures of parental involvement that reflect parental beliefs, perceptions, feelings, and attitudes may be better captured by parent-reports than independently observed behaviours (Palkovitz, 2019; Smith, 2011). We also modelled paternal involvement items across several time points in childhood, arguably painting a more fine-grained picture of paternal involvement compared to any one-off assessment through direct/independent observations. Nevertheless, this finding too should be interpreted with caution. Finally, the majority of participants in this study were of White ethnicity recruited within a specific region of the UK over two decades ago, which may limit the generalisability of our findings to other ethnic groups, geographic regions and sociodemographic circumstances (Fraser et al., 2013).

### Conclusions and Implications

Our findings emphasise the importance of the widely articulated, but rarely adopted, clinical stance to consider both maternal and paternal mental health when one parent presents with depression. Recognising and addressing paternal PND will support fathers and improve their ability to support their partners and children. The findings also highlight the importance of involving fathers in the treatment of maternal depression through partner-assisted therapies (e.g., interpersonal counselling; Brandon et al., 2012; Ingram et al., 2019), as well as interventions that address the importance of paternal involvement and caregiving directly via education and video-feedback (Lawrence et al., 2013; Olhaberry et al., 2019). Father-child conflict played a role in transmitting the negative effects of maternal PND on offspring development, even after accounting for higher levels of paternal PND. Should these effects be found to be causal in future research, targeted interventions to reduce father-child conflict may contribute to reducing intergenerational transmission of mental health risks in offspring of mothers affected by depression. Nonetheless, it is important to emphasise that maternal PND was strongly associated with adverse offspring development even in the context of paternal involvement, suggesting that addressing maternal PND remains a key factor in reducing adverse offspring outcomes.

The mother-child relationship is a cornerstone of developmental research, often at the expense of a wider family systems perspective, which explicitly acknowledges the father-child relationship as a crucial sub-system, integrated and influential in the context of the higher-order family system (Cowan & Cowan, 2006). This has important implications for policy and practice that should encourage and facilitate father involvement in interventions designed to improve offspring outcomes, without losing sight of the importance of treating mothers. Policies that facilitate and optimise father involvement in the context of maternal PND may provide an opportunity to improve family health and child well-being.

## Supplementary Material

Supplementary material

## Figures and Tables

**Table 1:** Characteristics of the sample and offspring total difficulties mean score at age 7 years by the exposure status (maternal PND at 8 weeks dichotomised at a cut-off ≥13)

Exposure status: N (%)	Maternal postnatal depression (8 weeks)	
	No	Yes
	10,383 (89.9)	1,170 (10.1)
	n (%)	n (%)
*Maternal educational attainment*		
A-Level/Degree	5,719 (89.8)	653 (10.2)
Minimal education/None/O-Level	3,718 (91.4)	349 (8.6)
Chi^2^, p-value		7.95, 0.005
*Financial difficulties*		
No financial difficulties	8,183 (91.8)	733 (8.2)
Financial difficulties	1,934 (82.9)	399 (17.1)
Chi^2^, p-value		161. 16, <0.001
*Child sex*		
Male	5,353 (89.8)	610 (10.2)
Female	5,030 (90.0)	560 (10.0)
Chi^2^, p-value		0.14, 0.706
*Home ownership*		
Owned/mortgaged	7,546 (91.9)	662 (8.1)
Private/council rented	1,692 (84.5)	310 (15.5)
Chi^2^, p-value		102.85, <0.001
*Marital status*		
Never married	2,181 (85.9)	359 (14.1)
Married	7,886 (91.2)	760 (8.8)
Chi^2^, p-value		62.27, <0.001
*Maternal age, mean (SD)*	27.6 (4.8)	27.2 (5.2)
ANOVA, p-value		2.88, 0.004
*Offspring total difficulties score at 7 years, mean (SD)*	7.3 (4.7)	9.8 (5.6)
ANOVA, p-value		-13.58, <0.001
*Parental antenatal conflict, mean (SD)*	9.2 (1.7)	10.1 (1.9)
ANOVA, p-value		16.14, <0.001

*Note*: p-values based on Pearson’s Chi-square (Chi^2^) test of association between maternal PND and categorical variables, and ANOVA for differences in means for continuous variables; sample sizes vary due to the differences in data availability on socioeconomic, child, parental and familial characteristics.

**Table 2:** **Model 1:** Associations between maternal PND (8 weeks), offspring development (7 years) and dimensions of paternal involvement in the model adjusted for child PGS and all antenatal baseline confounders (n=3,434)

Mediator: Paternal involvement	Exposure: Maternal PND (8 weeks)		Outcome: Offspring development (7 years)	
	Fully adjusted model estimates ^a^ (n=3,434)			
	*B* [95% CI]	P-value	*B* [95% CI]	P-value
**Child-focused dimensions of paternal involvement**				
*Paternal parenting confidence*	-0.049 [-0.061, -0.037]	≤0.001	-0.597 [-0.789, -0.404]	≤0.001
*Paternal conflictual relationship with child*	-0.053 [-0.063, -0.043]	≤0.001	-0.779 [-0.961, -0.597]	≤0.001
*Paternal enjoyment and warmth*	-0.032 [-0.042, -0.022]	≤0.001	-0.379 [-0.551, -0.206]	≤0.001
*Paternal involvement in childcare*	0.007 [-0.003, 0.017]	0.158	-0.043 [-0.237, 0.151]	0.664
*Paternal beliefs regarding caregiving*	-0.009 [-0.234, 0.216]	0.132	-0.019 [-0.244, 0.206]	0.872
*Paternal worries about child*	-0.027 [-0.039, -0.015]	≤0.001	-0.277 [-0.479, -0.075]	0.007
**Mother-influenced dimensions of paternal involvement**				
*Paternal help with household tasks*	0.001 [-0.010, 0.011]	0.932	-0.016 [-0.186, 0.154]	0.854
*Paternal perception of*	-0.040	≤0.001	-0.038	0.749
*maternal ‘gatekeeping’*	[-0.054, -0.026]		[-0.273, 0.197]	
*Paternal beliefs regarding mother-father relationship*	-0.030 [-0.042, -0.018]	≤0.001	-0.040 [-0.234, 0.154]	0.689
*Paternal beliefs regarding employment and parenthood*	-0.031 [-0.041, -0.021]	≤0.001	-0.224 [-0.391, -0.057]	0.008

a Effect size are regression coefficients (*β* unstandardised) in the fully adjusted models: adjusted for child PGS, gender and antenatal baseline confounders (financial problems, homeownership status, maternal age and education, parental conflict, marital status). *Maternal PND: maternal postnatal depression; Child PGS: child polygenic score for neuroticism*.

**Table 3:** **Model 2:** Associations between maternal PND (8 weeks), offspring development (7 years), paternal PND (8 months), and dimensions of paternal involvement in the model adjusted for child PGS, gender, antenatal baseline confounders, and paternal PND as an intermediate confounder (n=3,434)

Mediator: Paternal involvement	Exposure: Maternal PND (8 weeks)		Outcome: Offspring development (7 years)		Intermediate confounder: Paternal PND (8 months)	
	Fully adjusted model estimates ^a^ (n=3,434)					
	*B* [95% CI]	P-value	*B* [95% CI]	P-value	*B* [95% CI]	P-value
**Child-focused dimensions of paternal involvement**						
*Paternal parenting confidence*	0.029 [0.004, 0.054]	0.027	-0.309 [-0.732, 0.114]	0.153	-0.812 [-0.935, -0.689]	<0.001
*Paternal conflictual relationship with child*	0.034 [0.007, 0.061]	0.016	-0.546 [-0.998, -0.093]	0.018	-0.975 [-1.124, -0.826]	≤0.001
*Paternal enjoyment and warmth*	0.055 [0.031, 0.078]	≤0.001	0.072 [-0.316, 0.460]	0.718	-0.784 [-0.884, -0.684]	≤0.001
*Paternal involvement in childcare*	0.029 [0.017, 0.041]	≤0.001	0.099 [-0.110, 0.310]	0.351	-0.163 [-0.200, -0.128]	≤0.001
*Paternal beliefs regarding caregiving*	0.004 [-0.010, 0.018]	0.563	0.064 [-0.173, 0.301]	0.595	-0.102 [-0.141, -0.063]	≤0.001
*Paternal worries about child*	-0.011 [-0.023, 0.001]	0.073	-0.168 [-0.034, 0.370]	0.104	-0.117 [-0.150, -0.084]	≤0.001
*Maternal postnatal depression (8 weeks)*					0.139 [0.115, 0.162]	<0.001
*Offspring development (7 years)*					-0.410 [-0.533, 0.713]	0.475
**Mother-influenced dimensions of paternal involvement**						
*Paternal help with household tasks*	0.009 [-0.001, 0.019]	0.070	0.039 [-0.135, 0.213]	0.662	-0.061 [-0.083, -0.039]	≤0.001
*Paternal perception of maternal ‘gatekeeping’*	-0.003 [-0.021, 0.015]	0.771	0.216 [-0.109, 0.541]	0.192	-0.366 [-0.427, -0.305]	≤0.001
*Paternal beliefs regarding mother-father relationship*	0.007 [-0.013, 0.027]	0.508	0.295 [-0.056, 0.646]	0.100	-0.416 [-0.494, -0.338]	≤0.001
*Paternal beliefs regarding employment and parenthood*	-0.010 [-0.019, -0.001]	0.047	-0.097 [-0.281, 0.087]	0.300	-0.169 [-0.194, -0.143]	≤0.001
*Maternal postnatal depression (8 weeks)*					0.139 [0.115, 0.162]	<0.001
*Offspring development (7 years)*					0.337 [0.055, 0.619]	0.019

a Effect size are regression coefficients *(fi* unstandardised) in the fully adjusted models: adjusted for child PGS, gender and antenatal baseline confounders (financial problems, homeownership status, maternal age and education, parental conflict, marital status). Maternal PND: maternal postnatal depression; paternal PND: paternal postnatal depression; Child PGS: child polygenic score for neuroticism.

**Table 4:** Estimates of direct and mediated effects in the unadjusted model and models adjusted for child PGS, gender, antenatal baseline confounders, and paternal PND as an intermediate confounder in complete sample (n=3,434)

Effect Size ^1^	Unadjusted model		Model estimates (n=3,434) Model 1		Model 2	
	*B* [95% CI]	P-value	*B* [95% CI]	P-value	*B* [95% CI]	P-value
**Child-focused dimensions of paternal involvement**						
1. Total indirect effect^*^	0.104 [0.075, 0.133]	≤0.001	0.090 [0.063, 0.117]	≤0.001	0.082 [-0.028, 0.192]	0.144
2. Direct effect^**^	0.137 [0.094, 0.180]	≤0.001	0.120 [0.077, 0.163]	≤0.001	0.185 [0.110, 0.273]	≤0.001
3. Total effect^***^	0.241 [0.204, 0.278]	≤0.001	0.210 [0.171, 0.250]	≤0.001	0.267 [0.077, 0.457]	≤0.001
*4. Specific indirect effects*						
*Paternal parenting confidence*	0.031 [0.019, 0.043]	≤0.001	0.030 [0.018, 0.042]	≤0.001	0.026 [-0.015, 0.067]	0.225
*Paternal conflictual relationship with child*	0.049 [0.035, 0.063]	≤0.001	0.041 [0.029, 0.053]	≤0.001	0.055 [0.001, 0.110]	0.046
*Paternal enjoyment and warmth*	0.014 [0.006, 0.022]	≤0.001	0.012 [0.004, 0.020]	0.001	-0.004 [-0.028, 0.020]	0.752
*Paternal involvement in childcare*	0.001 [-0.001, 0.003]	0.874	0.001 [-0.001, 0.003]	0.746	0.001 [-0.001, 0.003]	0.594
*Paternal beliefs regarding caregiving*	0.001 [-0.001, 0.003]	0.843	0.001 [-0.001, 0.003]	0.899	0.001 [-0.001, 0.003]	0.690
*Paternal worries about child*	0.010 [0.002, 0.018]	0.005	0.007 [0.001, 0.013]	0.028	0.005 [-0.001, 0.011]	0.149
**Mother-influenced dimensions of paternal involvement**						
*1. Total indirect effect* ^*^	0.031 [0.007, 0.055]	0.008	0.010 [-0.008, 0.028]	0.291	-0.023 [-0.066, 0.020]	0.282
2. Direct effect^**^	0.210 [0.167, 0.253]	≤0.001	0.200 [0.157, 0.243]	≤0.001	0.186 [0.139, 0.233]	≤0.001
3. Total effect^***^	0.241 [0.204, 0.278]	≤0.001	0.210 [0.171, 0.250]	≤0.001	0.163 [0.092, 0.234]	≤0.001
*4. Specific indirect effects Paternal help with household tasks*	0.001 [-0.001, 0.003]	0.985	0.001 [-0.001, 0.003]	0.989	0.001 [-0.001, 0.003]	0.973
*Paternal perception of maternal ‘gatekeeping’*	0.012 [-0.002, 0.026]	0.098	0.002 [-0.010, 0.014]	0.799	-0.012 [-0.036, 0.012]	0.323
*Paternal beliefs regarding mother-father relationship and parenting*	0.006 [-0.004, 0.016]	0.217	0.001 [-0.001, 0.009]	0.737	-0.015 [-0.039, 0.009]	0.224
*Paternal beliefs regarding employment and parenthood*	0.013 [0.005, 0.021]	0.002	0.007 [0.001, 0.013]	0.032	0.003 [-0.005, 0.011]	0.370

1 Effect size are unadjusted and adjusted bootstrapped (n=1,000) regression coefficients (*B* unstandardised); unadjusted model (exposure, outcome and mediators only); Model 1: adjusted for child PGS and antenatal baseline confounders (child gender, financial problems, homeownership status, maternal age and education, parental conflict, marital status); Model 2: further adjusted for paternal PND at 8 months as an intermediate confounder.

**Model 1:** ^*^Total indirect effect = maternal PND->aspects of paternal involvement->offspring emotional and behavioural development; ^**^Direct effect = maternal PND-> offspring emotional and behavioural development; ^***^Total effect = total indirect effect + direct effect.

**Model 2:** ^*^Total indirect effect = maternal PND->aspects of paternal involvement->offspring emotional and behavioural development + maternal PND->paternal PND->aspects of paternal involvement -> offspring emotional and behavioural development; ^**^Direct effect = maternal PND-> offspring emotional and behavioural development + maternal PND->paternal PND->offspring emotional and behavioural development; ^***^Total effect = total indirect effect + direct effect. Maternal PND: maternal postnatal depression; PGS: Polygenic Score for Neuroticism; Paternal PND: paternal postnatal depression.
