# Supplementary material for: Maternal postnatal depressive symptoms and offspring emotional and behavioural development at age 7 years in a UK-birth cohort: the role of paternal involvement
Source: Dev Psychol. Author manuscript; Available in PMC 2023 Sep 6. (PMC7615033; doi:10.1037/dev0001482)
Supplement: Supplementary material [file EMS184919-supplement-Supplementary_material.pdf]

Culpin, I., Hammerton, G. L., Stein, A., Bornstein, M. H., Tiemeier, H., Cadman, T., Fredriksen, E., Evans, J., Miller, T., Dermott, E., Heron, J. E., Sallis, H. M., & Pearson, R. M. (Accepted/In press). Maternal postnatal depression and offspring emotional and behavioural development at age 7 years in a UK-birth cohort: the role of paternal involvement. *Developmental Psychology*. https://doi.org/10.1037/dev0001482

Peer reviewed version

Link to published version (if available):
10.1037/dev0001482

Link to publication record in Explore Bristol Research
PDF-document

This is the accepted author manuscript (AAM). The final published version (version of record) is available online via Wiley at https://doi.org/10.1037/dev0001482.Please refer to any applicable terms of use of the publisher.

University of Bristol - Explore Bristol Research

General rights

This document is made available in accordance with publisher policies. Please cite only the published version using the reference above. Full terms of use are available: http://www.bristol.ac.uk/red/research-policy/pure/user-guides/ebr-terms/

Maternal postnatal depressive symptoms and offspring emotional and behavioural development at age 7 years in a UK-birth cohort: the role of paternal involvement

Supplemental Material

Method

Measures

Mediators: Child-Focused and Mother-Influenced Dimensions of Paternal Involvement

Potential parenting items (>150) were extracted from paternal self-reported questionnaires administered on 5 occasions postnatally (8 weeks and 8 months, 1 year 9 months, 2 years 9 months, 3 years 11 months). Extracted items captured various dimensions of paternal involvement, which were initially double-rated and independently assigned into theoretical dimensions by three researchers (IC, RP and TC). This iterative process was followed by larger research group discussions with significant input from experts in early child development and parenting (AS and MB). Individual items fell into two theoretically distinct sources of paternal involvement: (1) *child-focused* paternal involvement capturing behavioural, affective and cognitive dimensions directed at the child; and (2) *mother-influenced* paternal involvement with the child through the lens of maternal expectations, mother-father relationship and indirect material care through support of the mother. The mother-influenced dimensions of paternal involvement acknowledge those aspects that are done for the child but do not entail interactions with the child, as well as the impact of inter-parental relationship on parent-child relationship (Cabrera et al., 2014; Pleck, 2012). It has been argued that men's engagement and interactions with their children need to be understood through the lens of maternal influences (Marsiglio et al., 2000; Palkovitz, 2019). Arguablyfathers are more involved when their female partners have supportive attitudes

regarding paternal involvement, and less involved when inter-parental relationship is characterised by underlying conflict, marital dissatisfaction and maternal ‘gatekeeping’ (maternal beliefs and practices that discourage or facilitate paternal involvement; Feldman, 2000; Miller, 2018). Evidence suggests that such influences may be more pronounced for paternal rather maternal involvement and parenting behaviours (Belsky et al., 1991; Schacht et al., 2009). Supporting the mother through sharing household tasks and responsibilities is another important dimension of paternal mother-influenced involvement (Cabrera et al., 2014). It may not directly land itself into an aspect of involvement, however, variations in this behaviour may have important implications for the child (Feldman, 2000; Pleck, 2012).

The final factors capturing child-focused paternal involvement (Table S1) encompassed: behavioural (e.g., direct involvement in caregiving), affective (e.g., quality of father-child relationship, such as enjoyment and warmth, conflictual relationship with and worries about the child), and cognitive (e.g., paternal parenting confidence and beliefs regarding caregiving) dimensions. The final factors capturing mother-influenced paternal involvement (Table S2) included: paternal perceptions of maternal beliefs or practices that discourage or facilitate his involvement in childcare (e.g., maternal ‘gatekeeping’), paternal help with household tasks and responsibilities (e.g., preparing meals and cleaning home), paternal beliefs regarding mother-father relationship and its impact on parenting (e.g., partner feels hurt by attention mother gives child) and maternal expectations around employment and parenthood (e.g., mother expects partner to take child after work).

Potential Baseline Confounders: Child Polygenic Score for Neuroticism, Socioeconomic, Parental and Family Characteristics

Analyses were adjusted for child polygenic score (PGS) for neuroticism. Genotyped data were available for 8,237 children in the ALSPAC cohort. Previously, 116 independent

variants robustly associated with neuroticism have been identified (Luciano et al., 2018). Of these original variants, 109 were available in the ALSPAC participants with the genetic data. Neuroticism PGS scores were calculated as the sum of the number of copies of each effect allele carried by an individual (ranging from 0 to 2 for each SNP), multiplied by the effect estimate identified in the original GWAS. These weighted standardised neuroticism PGS sum scores were included in the analyses.

Potential Intermediate Confounder: Paternal Postnatal Depression

Analyses were adjusted for paternal postnatal depression (PND) as a potential exposure induced intermediate confounder of the mediator-outcome association (Cole & Hernán, 2002; Sheikh et al., 2016). Paternal PND (self-reported depressive symptoms, no a clinical diagnosis) was assessed using the Edinburgh Postnatal Depression Scale (EPDS; Cox et al., 1987) administered to fathers at 8 months postnatally. The EPDS is a 10-item self-reported depression questionnaire, validated and used extensively to screen for depression during the perinatal period in women and men (Massoudi et al., 2013; Matthey et al., 2001). Individual items were summed-up to derive a continuous measure (score range 0-24, with higher scores indicating more severe depressive symptoms) to capture the full variation in depressive symptoms.

Statistical analysis

Latent Factor Models

Individual parenting items were loaded onto the hypothesised parenting dimensions and modelled using Confirmatory Factor Analyses (CFA). To identify the model, the mean of the latent factors was fixed to ‘0’ and their variance to ‘1’. The majority of individual items loaded strongly onto the factors that they were initially theoretically assigned to. However,

some items were moved between the factors if modification indices from the initial model indicated that items would be a better fit on the alternative factor, and this made theoretical sense following discussions by the research group. Additional correlations between similar items that were collected at different time points were added into the models to account for shared variance related to time and the repeated nature of the questions. Derived factors, items and factor loadings for each of the measurement models (child-focused and mother-influenced paternal involvement) are presented in Tables S1-S2.

Direct and Mediated Effects

First, we estimated the unadjusted models composed of exposure, outcome and mediators only. Second, we estimated models adjusted for child PGS and all antenatal baseline confounders (Model 1), and further adjusted for paternal PND (8 months) as a possible intermediate confounder of the mediator-outcome association (Model 2), which is also a mediator of the exposure -> mediator association. Thus, our path-specific effects of interest were: (i) Model 1: the indirect effect composed of maternal PND -> paternal involvement -> offspring emotional and behavioural development pathways, and the direct effect, which is the pathway from maternal PND to offspring emotional and behavioural development; (ii) Model 2: the indirect effect composed of a combination of pathways maternal PND -> paternal PND-> paternal involvement -> offspring emotional and behavioural development and maternal PND -> paternal involvement -> offspring emotional and behavioural development pathways, and the direct effect, which is a combination of maternal PND -> paternal PND -> offspring emotional and behavioural development pathways and maternal PND -> offspring emotional and behavioural development pathway. Figure 4a-b represent pathways that constitute total indirect (bold lines) and direct (dashed lines) pathways. We used MODEL INDIRECT (Model 1) and MODEL CONSTRAINT

(Model 2) commands in M*plus* v.8.3 (Muthén & Muthén, 2015) to estimate direct, total indirect and specific indirect effects with a WLSMV estimator to model continuous and categorical variables.

Missing Data: Multiple Imputation

We imputed missing data due to loss to follow-up because ignoring those with missing data may result in bias by assuming that data are Missing Completely at Random (MCAR; Sterne et al., 2009). We repeated direct and mediated effects analyses on the imputed datasets to account for the loss to follow-up. We used Multivariable Imputation by Chained Equations (MICE; Royston & White, 2011) to impute missing data in exposure, outcome and confounders (except child neuroticism PGS; n=6,029) using the *ice* command in Stata v.15.1/MP (Stata.Corp., Texas, USA). We did not impute child neuroticism PGS from the phenotype data due to restrictions associated with quality control and participant consent. ALSPAC provides a wealth of rich, prospectively collected data on a range of sociodemographic, mental health and developmental variables, which enabled us to account for missing data in exposure, outcome and confounders, as well as factors that explain missingness, validating the Missing-At-Random assumption (MAR; White et al., 2011). However, the decision was taken not to impute mediators (child-focused and mother-influenced dimensions of paternal involvement) as there are no sufficient auxiliary data on fathers' parenting in the ALSPAC cohort to justify the plausibility of MAR assumption. The imputation model was fully compatible with the main analyses. Using binary, ordinal logistic and linear regression models as appropriate, 50 imputed datasets by 10 cycles of regression switching were generated. Monte-Carlo errors were less than 10% of the standard error and FMI values were no larger than 0.5, suggesting that 50 imputed datasets were sufficient (White et al., 2011). Each imputation model contained all variables in the substantive

analyses along with over 40 auxiliary variables pertaining to maternal and paternal characteristics (e.g., paternal age) and psychopathology, offspring development and mental health, as well maternal and paternal indices of socioeconomic adversity. MICE imputations were carried out in Stata v.15.1/MP (Stata.Corp., Texas, USA). The imputed datasets were exported into M*plus* v.8.3 (Muthén & Muthén, 2015) to estimate direct and indirect (mediated) pathways using MODEL CONSTRAINT command.

Results S1

Paternal Involvement Factors

Full details of six factors capturing child-focused and four factors capturing mother-influenced aspects of paternal involvement are provided below.

Child-Focused Paternal Involvement

Factor 1 Paternal parenting confidence: 11 items relating to paternal feelings of confidence in the parenting role and perceptions of the ability to engage effectively in parenting behaviours (e.g., ‘partner feels confident with child’, ‘partner unsure if doing the right thing’, ‘partner happy with the way he brings up child’) were extracted from paternal self-reported questionnaires administered at 8 weeks, 8 months, 1 year 9 months and 2 years 9 months. Higher factor scores represented higher levels of paternal parenting confidence.

Factor 2 Paternal conflictual relationship with child: 19 items relating to conflict, harsh disciplining and irritation with the child (e.g., ‘child gets on partner’s nerves’, ‘partner dislikes mess surrounding child’, ‘smacking is the best way to discipline child’) were extracted from paternal self-reported questionnaires administered at 8 weeks, 8 months, 1 year 9 months and 2 years 9 months. Higher factor scores signified lower levels of conflictual parent-child relationship, irritation with the child and less harshness in paternal disciplining.

Factor 3 Paternal enjoyment and warmth: 27 items relating to feelings of enjoyment, affection, love and warmth toward the child (e.g., 'partner enjoys child', 'partner feels very close to child', 'child gives great joy') were extracted from paternal self-reported questionnaires administered at 8 weeks, 8 months, 1 year 9 months, 2 years 9 months and 3 years 11 months. Higher factor scores represented more paternal enjoyment, affection and warmth toward the child.

Factor 4 Paternal involvement in childcare: 8 items describing frequency of paternal involvement in various aspects of childcare (e.g., 'frequency partner feeds child per week', 'frequency partner puts child to bed per week', 'frequency partner bathes child per week') were extracted from paternal self-reported questionnaires administered at 8 weeks and 8 months. Higher factor scores represented higher frequency of paternal involvement in childcare.

Factor 5 Paternal worries about child: 4 items pertaining to paternal worries about the child (e.g., 'partner worries about child when at work', 'partner anxious if someone else looks after child', 'partner worries about study child when at work') were extracted from paternal self-reported questionnaires administered at 1 year 9 months and 2 years 9 months. Higher factor scores represented lower levels of paternal worries about the child.

Factor 6 Paternal beliefs regarding caregiving: 6 items relating to paternal parenting principles and practices related to structure, regularity and routine in infant care (e.g., 'babies should fit into parents' routine', 'babies should be fed when hungry') and attunement, i.e. responsiveness to infant cues (e.g., 'babies should be picked up when cry') were extracted from paternal self-reported questionnaires administered at 8 months and 1 year 9 months. Higher factor scores represented more paternal appreciation of regular routine and higher levels of attunement and responsiveness to child's cues.

Mother-Focused Paternal Involvement

Factor 1 Paternal help with household tasks: 12 items related to various aspects of paternal help with household tasks (e.g., 'partner helped with cleaning home since birth', 'partner helped with housework since birth', 'partner gave help with preparing meals') were extracted from paternal self-reported questionnaires administered at 8 weeks and 8 months. Higher factor scores represented higher levels of paternal help with household tasks since the child was born.

Factor 2 Paternal perception of maternal 'gatekeeping': 7 items relating to paternal perceptions of maternal beliefs and behaviours that encourage or hinder paternal involvement in childcare, i.e., maternal 'gatekeeping', (e.g., 'mother excludes partner from childcare', 'partner always getting under mother's feet, 'mother dislikes partner being involved with childcare') were extracted from paternal self-reported questionnaires administered at 8 weeks. Higher factor scores indicated higher levels of paternal perception of being supported by the mother and included in childcare, i.e. less maternal 'gatekeeping'.

Factor 3 Paternal beliefs regarding mother-father relationship and parenting: 3 items relating to paternal beliefs regarding the impact of having a child on father-mother relationship (e.g., 'parenthood has made partner and mother closer', 'mother no longer gives partner attention') were extracted from paternal self-reported questionnaires administered at 8 weeks. Higher factor scores represented paternal beliefs more concordant with positive changes in the nature of mother-father relationship following the birth of the child.

Factor 4 Paternal attitudes to employment and parenthood: 5 items relating to paternal beliefs regarding maternal expectations around employment, childcare and paternal difficulties to manage childcare and employment (e.g., 'mother expects partner to take child

after work', 'partner too tired to take child after work', 'partner finds it hard to cope with child after work') were extracted from paternal self-reported questionnaires at 8 weeks and 2 years 9 months. Higher factor scores indicated paternal beliefs concordant with less maternal pressure to look after child after work and less difficulties with managing childcare and employment.

Associations Between Child-Focused and Mother-Influenced Dimensions of Paternal Involvement

Paternal parenting confidence was strongly associated with less conflictual father-child relationship, more paternal enjoyment and involvement in childcare and less paternal worries about the child (Table S3). Conflictual father-child relationship was associated with less enjoyment and warmth, less involvement in childcare, less appreciation of structure and more worries about the child. Paternal enjoyment and warmth were associated with more involvement in childcare, more paternal appreciation of structure and higher levels of attunement to child's cues. Paternal worries about the child were associated with less appreciation of structure and less involvement in childcare. Paternal beliefs more concordant with the positive impact of having a child on mother-father relationship were associated with higher levels of paternal help with household tasks and less pressure to manage childcare and employment. Paternal perception of maternal support and inclusion in childcare were associated with higher levels of paternal involvement in household tasks, better ability to manage employment and childcare, and more positive feelings on the changing nature of mother-father relationship following the birth of the child.

Associations Between Maternal PND, Child-Focused and Mother-Influenced Dimensions of Paternal Involvement and Offspring Emotional and Behavioural Development

Full description of estimates for Model 1 is provided below, with full description of estimates for Model 2 provided in the main manuscript.

Exposure (Maternal PND) – Mediator (Paternal Involvement) Associations

In Model 1 (adjusted for child PGS and antenatal baseline confounders; Table 2) maternal PND at 8 weeks was strongly associated with less paternal parenting confidence (β=-0.049, 95% CI: -0.061, -0.037, p≤0.001), more father-child conflict (β=-0.053, 95% CI: -0.063, -0.043, p≤0.001), less paternal enjoyment and warmth (β=-0.032, 95% CI: -0.042, -0.022, p≤0.001), more paternal worries about the child (β=-0.027, 95% CI: -0.039, -0.015, p≤0.001), higher levels of perceived maternal 'gatekeeping' (β=-0.040, 95% CI: -0.054, -0.026, p≤0.001), more negative feelings regarding the impact the birth of the child have had on mother-father relationship (β=-0.030, 95% CI: -0.042, -0.018, p≤0.001), and paternal feelings of more pressure to look after the child after work and more struggles to manage childcare and employment (β=-0.031, 95% CI: -0.041, -0.021, p≤0.001; Table 2).

Maternal PND also had an indirect effect on paternal engagement via paternal PND (Table 3). Specifically, maternal PND was strongly associated with paternal PND (β=0.139, 95% CI: 0.115, 0.162, p≤0.001), which, in turn, was strongly associated with less paternal parenting confidence (β=-0.812, 95% CI: -0.935, -0.689, p≤0.001), less enjoyment and warmth (β=-0.784, 95% CI: -0.884, -0.684, p≤0.001), less involvement in childcare (β=-0.163, 95% CI: -0.200, -0.128, p≤0.001) and appreciation of regular routine (β=-0.102, 95% CI: -0.141, -0.063, p≤0.001), more father-child conflict (β=-0.975, 95% CI: -1.124, -0.826, p≤0.001) and more paternal worries about the child (β=-0.117, 95% CI: -0.150, -0.084, p≤0.001), less paternal help with household tasks (β=-0.061, 95% CI: -0.083, -0.039, p≤0.001), higher levels of perceived maternal 'gatekeeping' (β=-0.366, 95% CI: -0.427, -0.305, p≤0.001), more negative feelings regarding the impact the birth of the child have had

on mother-father relationship (β=-0.416, 95% CI: -0.494, -0.338, p≤0.001), and paternal feelings of more pressure to look after the child after work and more struggles to manage childcare and employment (β=-0.169, 95% CI: -0.194, -0.143, p≤0.001; Table 3).

Mediator (Paternal Involvement) – Outcome (Offspring Emotional and Behavioural Development) Associations

In Model 1, lower levels of paternal parenting confidence (β=-0.597, 95% CI: -0.789, -0.404, p≤0.001), warmth and enjoyment (β=-0.379, 95% CI: -0.551, -0.206, p≤0.001), higher levels of father-child conflict (β=-0.779, 95% CI: -0.961, -0.597, p≤0.001) and worries about the child (β=-0.277, 95% CI: -0.479, -0.075, p=0.007) were associated with higher risk of offspring emotional and behavioural difficulties at age 7 years (Table 2). Out of all mother-influenced dimensions of paternal involvement, only paternal feelings of more pressure to look after the child after work and more struggles to manage childcare and employment were associated with increased risk of offspring emotional and behavioural difficulties (β=-0.224, 95% CI: -0.391, -0.057, p=0.008).

Direct and Mediated Effects

In Model 1 (adjusted for child PGS and antenatal baseline confounders; Table 4), there was evidence of total indirect effect from early maternal PND to offspring development at age 7 years through the combination of all parenting factors capturing child-focused (β=0.090, 95%CIs: 0.063, 0.117, p≤0.001), but not mother-influenced dimensions of paternal involvement (β=0.010, 95%CIs: -0.008, 0.028, p=0.291). There was strong evidence of total and direct effects from maternal PND to offspring emotional and behavioural development at age 7 years in models capturing both child-focused (total effect: β=0.210, 95%CIs: 0.171, 0.250, p≤0.001; direct effect: β=0.120, 95%CIs: 0.077, 0.163, p≤0.001) and mother-

influenced (total effect: β=0.210, 95%CIs: 0.171, 0.250, p≤0.001; direct effect: β=0.200, 95%CIs: 0.157, 0.243, p≤0.001) paternal involvement. Amongst child-focused dimensions of paternal involvement, there was evidence for specific indirect effects through paternal parenting confidence (β=0.030, 95%CIs: 0.018, 0.042, p≤0.001), paternal conflictual relationship with the child (β=0.041, 95%CIs: 0.029, 0.053, p≤0.001), paternal enjoyment and warmth (β=0.012, 95%CIs: 0.004, 0.020, p=0.001), and paternal worries about the child (β=0.007, 95%CIs: 0.001, 0.013, p=0.028), but not paternal involvement in childcare and endorsement of caregiving structure and attunement to child's cues. There was only some evidence for specific indirect effect through paternal attitudes to employment and parenthood (β=0.007, 95%CIs: 0.001, 0.013, p=0.032), although 95% CIs were wide, amongst mother-influenced dimensions of paternal involvement.

Sensitivity Analyses: Missing Data

We repeated direct and mediated effects analyses on imputed datasets to examine the impact of response attrition on our findings. Characteristics of the sample by the completeness of the data are presented in Table S4. Participants comprising our analytical sample were from a higher socioeconomic background as indexed by lower proportion of those reporting financial difficulties and higher proportion of those reporting residing owned/mortgaged accommodation and married marital status compared to the original ALSPAC sample. The results from the analyses using the imputed data supported our findings resulting in the similar pattern of results and over-arching conclusions as our main findings in the complete case analyses (Table S5) with one notable difference. In Model 2, there was stronger evidence for the specific indirect effect through paternal conflictual relationship with the child (β=0.042, 95%CIs: 0.005, 0.079, p=0.028), with some emerging evidence for a specific indirect effect through paternal worries about the child although the

95%CIs were wide (β=0.006, 95%CI 0.001, 0.012, p=0.037) compared to complete case analyses (Table 4).

References

Belsky, J., Youngblade, L., Rovine, M., & Volling, B. (1991). Patterns of marital change and parent-child interaction. *Journal of Marriage and the Family*, 487-498. https://doi.org/10.2307/352914

Cabrera, N. J., Fitzgerald, H. E., Bradley, R. H., & Roggman, L. (2014). The ecology of father-child relationships: An expanded model. *Journal of Family Theory & Review*, *6*(4), 336-354. https://doi.org/10.1111/jftr.12054

Cole, S. R., & Hernán, M. A. (2002). Fallibility in estimating direct effects. *International Journal of Epidemiology*, *31*(1), 163-165. https://doi.org/10.1093/ije/31.1.163

Cox, J. L., Holden, J. M., & Sagovsky, R. (1987). Detection of postnatal depression: development of the 10-item Edinburgh Postnatal Depression Scale. *British Journal of Psychiatry*, *150*(6), 782-786. https://doi.org/10.1192/bjp.150.6.782

Feldman, R. (2000). Parents' convergence on sharing and marital satisfaction, father involvement, and parent–child relationship at the transition to parenthood. *Infant Mental Health Journal: Official Publication of The World Association for Infant Mental Health*, *21*(3), 176-191. https://doi.org/10.1002/1097-0355(200007)21:3<176::AID-IMHJ3>3.0.CO;2-4

Luciano, M., Hagenaars, S. P., Davies, G., Hill, W. D., Clarke, T. K., Shirali, M., Harris, S. E., Marioni, R. E., Liewald, D. C., Fawns-Ritchie, C., Adams, M. J., Howard, D. M., Lewis, C. M., Gale, C. R., McIntosh, A. M., & Deary, I. J. (2018). Association analysis in over 329,000 individuals identifies 116 independent variants influencing neuroticism. *Nature Genetics*, *50*(1), 6-11. https://doi.org/10.1038/s41588-017-0013-8

Marsiglio, W., Day, R. D., & Lamb, M. E. (2000). Exploring fatherhood diversity: Implications for conceptualizing father involvement. *Marriage & Family Review*, *29*(4), 269-293. https://doi.org/10.1300/J002v29n04_03

Massoudi, P., Hwang, C. P., & Wickberg, B. (2013). How well does the Edinburgh Postnatal Depression Scale identify depression and anxiety in fathers? A validation study in a population based Swedish sample. *Journal of affective disorders*, *149*(1-3), 67-74. https://doi.org/10.1016/j.jad.2013.01.005

Matthey, S., Barnett, B., Kavanagh, D. J., & Howie, P. (2001). Validation of the Edinburgh Postnatal Depression Scale for men, and comparison of item endorsement with their partners. *Journal of affective disorders*, *64*(2-3), 175-184. https://doi.org/10.1016/S0165-0327(00)00236-6

Miller, T. (2018). Paternal and maternal gatekeeping? Choreographing care. *Sociologica*, *12*, 25-35. https://doi.org/10.6092/issn.1971-8853/9083

Muthén, L. K., & Muthén, B. O. (2015). *Mplus User's Guide*, 7th ed. Muthén & Muthén: Los Angeles, CA.

Palkovitz, R. (2019). Expanding our focus from father involvement to father–child relationship quality. *Journal of Family Theory & Review*, *11*(4), 576-591. https://doi.org/10.1111/jftr.12352

Pleck, J. H. (2012). Integrating father involvement in parenting research. *Parenting*, *12*(2-3), 243-253. https://doi.org/10.1080/15295192.2012.683365

Royston, P., & White, I. R. (2011). Multiple imputation by chained equations (MICE): implementation in Stata. *Journal of Statistical Software*, *45*(4), 1-20.

Schacht, P. M., Cummings, E. M., & Davies, P. T. (2009). Fathering in family context and child adjustment: A longitudinal analysis. *Journal of Family Psychology*, *23*(6), 790. https://doi.org/10.1037/a0016741

Sheikh, M. A., Abelsen, B., & Olsen, J. A. (2016). Differential recall bias, intermediate confounding, and mediation analysis in life course epidemiology: an analytic framework with empirical example. *Frontiers in Psychology*, *7*, 1828. https://doi.org/10.3389/fpsyg.2016.01828

Sterne, J. A. C., White, I. R., Carlin, J. B., Royston, P., Carpenter, J. R. (2009). Multiple imputation for missing data in epidemiological and clinical research: potential and pitfalls. *BMJ*, 338, b2393-b2393. https://doi.org/10.1136/bmj.b2393

White, I. R., Royston, P., & Wood, A. M. (2011). Multiple imputation using chained equations: issues and guidance for practice. *Statistics in Medicine*, *30*(4), 377-99. https://doi.org/10.1002/sim.4067

Table S1. *Derived factors, items, standardised factor loadings and fit indices for the measurement model capturing child-focused dimensions of paternal involvement*

Item	Age at assessment/Factor	Item description	Factor loading	S.E.
**I**	**Paternal parenting confidence**			
1.	8 weeks	Partner feels confident with child	0.521	0.016
2.	8 weeks	Partner happy with the way he brings up child	0.657	0.015
3.	8 weeks	Partner regrets lack of experience with child	0.363	0.022
4.	8 weeks	Partner feels well-prepared for birth and childcare	0.403	0.015
5.	8 weeks	Having a baby is as expected	0.356	0.015
6.	8 months	Partner feels confident with child	0.711	0.021
7.	8 months	Partner unsure whether doing the right thing	0.436	0.017
8.	1 year 9 months	Partner afraid to be alone with toddler	0.478	0.055
9.	1 year 9 months	Partner sure doing the right thing for child	0.574	0.016
10.	2 years 9 months	Partner feels confident with child	0.694	0.021
11.	2 years 9 months	Partner feels constantly unsure if doing right thing for child	0.434	0.019
**II**	**Paternal conflictual relationship with child**			
1.	8 weeks	Partner so stressed at home it is a bad influence on child	0.557	0.017
2.	8 weeks	Partner regrets having child	0.688	0.022
3.	8 months	Partner dislikes mess surrounding child	0.532	0.014
4.	8 months	Partner finds child crying unbearable	0.564	0.014
5.	8 months	Partner feels they have no time to themselves	0.590	0.013
6.	1 year 9 months	Child whining can make parent want to hit	0.477	0.015
7.	1 year 9 months	Having young child is absolutely exhausting	0.451	0.014
8.	1 year 9 months	Smacking is the best way to discipline child	0.168	0.017
9.	1 year 9 months	Parent can feel exasperated calming child	0.366	0.015
10.	1 year 9 months	Child never gets on partner’s nerves	0.306	0.015
11.	1 year 9 months	Partner cannot bear it when child cries	0.364	0.016
12.	1 year 9 months	Partner feels desperate when child complains	0.548	0.015
13.	1 year 9 months	Child demands bring intense anger	0.555	0.017
14.	2 years 9 months	Partner dislikes mess surrounding child	0.550	0.015
15.	2 years 9 months	Partner cannot bear it when child cries	0.434	0.016
16.	2 years 9 months	Partner feels they have no time to themselves	0.556	0.015
17.	2 years 9 months	Partner often gets very irritated with child	0.454	0.020
18.	2 years 9 months	Partner has frequent battles of will with child	0.290	0.021
19.	2 years 9 months	Child gets on partner’s nerves	0.438	0.025
**III**	**Paternal enjoyment and warmth**			
1.	8 weeks	Partner is making a strong bond with child	0.515	0.013
2.	8 weeks	Partner feels guilty at not enjoying child	0.651	0.015
3.	8 weeks	Child made partner more fulfilled	0.486	0.013
4.	8 months	Partner enjoys child	0.657	0.014
5.	8 months	Partner preferred not to have had child	0.559	0.018
6.	8 months	Pleasure watching child develop	0.693	0.016
7.	8 months	Partner feels should enjoy child, but they are not	0.704	0.015

8.	8 months	Child made partner more fulfilled	0.550	0.012
9.	8 months	Partner feels children are fun	0.646	0.012
10.	8 months	Partner enjoys seeing child after work	0.639	0.016
11.	8 months	Partners feels having child made more fulfilled	0.541	0.016
12.	8 months	Talking to child is important	0.352	0.036
13.	8 months	Cuddling child is very important	0.375	0.037
14.	1 year 9 months	Children are fun	0.568	0.014
15.	1 year 9 months	Partner really loves child	0.708	0.020
16.	1 year 9 months	Partner glad they had child	0.670	0.016
17.	1 year 9 months	Partner feels great pleasure watching child grow	0.673	0.018
18.	1 year 9 months	Child gives great joy	0.705	0.016
19.	2 years 9 months	Partner really enjoys child	0.659	0.015
20.	2 years 9 months	Partner feels great pleasure watching child develop	0.730	0.017
21.	2 years 9 months	Partner feels should enjoy child, but they are not	0.604	0.017
22.	2 years 9 months	Partners feels having child made more fulfilled	0.597	0.014
23.	2 years 9 months	Partner enjoys seeing child after work	0.607	0.017
24.	3 years 11 months	Child makes partner feel very happy	0.286	0.062
25.	3 years 11 months	Partner feels very close to child	0.422	0.045
26.	3 years 11 months	Child is very affectionate to partner	0.384	0.037
27.	3 years 11 months	Cuddling is best way to calm child	0.643	0.015
**IV**	**Paternal involvement in childcare**			
1.	8 weeks	Frequency partner changes nappy per week	0.644	0.014
2.	8 weeks	Frequency partner bathes child per week	0.387	0.014
3.	8 weeks	Frequency partner plays with child per week	0.751	0.022
4.	8 weeks	Frequency partner walks child outside per week	0.220	0.014
5.	8 weeks	Frequency partner puts child to bed per week	0.593	0.013
6.	8 weeks	Frequency partner feeds child or helps at night per week	0.515	0.014
7.	8 weeks	Frequency partner feeds child per week	0.642	0.017
8.	8 months	Frequency partner gives help changing nappies	0.449	0.020
**V**	**Paternal worries about child**			
1.	1 year 9 months	Partner worries if child is eating enough	0.733	0.018
2.	1 year 9 months	Get child to eat right food makes partner anxious	0.935	0.021
3.	1 year 9 months	Partner anxious if someone else looks after child	0.372	0.017
4.	2 years 9 months	Partner worries about study child when at work	0.340	0.024
**VI**	**Paternal beliefs regarding caregiving**			
1.	8 months	Babies should be picked up when cry	0.482	0.030
2.	8 months	Babies should be fed when hungry	0.552	0.033
3.	8 months	Parents need to adapt lives to babies needs	0.320	0.026
4.	8 months	Baby should fit into parents' routine	0.143	0.026
5.	8 months	Babies should be stimulated to develop well	0.311	0.029
6.	1 year 9 months	Toddlers should be able to eat when they ask	0.207	0.042
	**Latent factor model fit indices**			
	Free parameters			299
	Root Mean Square Error of Approximation (RMSEA)			0.022 (95%CI 0.021 to 0.022)
	Comparative Fit Index (CFI)			0.904
	Tucker-Lewis Index (TLI)			0.899

Table S2. *Derived factors, items, standardised factor loadings and fit indices for the measurement model capturing mother-influenced dimensions of paternal involvement*

Item	Age at assessment/Factor	Item description	Factor loading	S.E.
**I**	**Paternal help with household tasks**			
1.	8 weeks	Partner helped with shopping since birth	0.518	0.011
2.	8 weeks	Partner helped with cleaning home since birth	0.591	0.011
3.	8 weeks	Partner helped with meal preparation since birth	0.599	0.013
4.	8 weeks	Partner helped with washing up since birth	0.687	0.011
5.	8 weeks	Partner helped with housework since birth	0.642	0.009
6.	8 weeks	Partner helped with cooking meals since birth	0.613	0.010
7.	8 weeks	Partner helped with clothes wash since birth	0.491	0.011
8.	8 months	Partner gave help with shopping	0.427	0.012
9.	8 months	Partner gave help with cleaning home	0.533	0.011
10.	8 months	Partner gave help preparing meals	0.688	0.011
11.	8 months	Partner gave help washing up	0.498	0.012
12.	8 months	Partner gave help washing clothes	0.414	0.012
**II**	**Paternal perception of maternal 'gatekeeping'**			
1.	8 weeks	Pressure on partner to be present at birth	0.240	0.023
2.	8 weeks	Mother excludes partner from childcare	0.660	0.020
3.	8 weeks	Partner feels mother does not trust him with childcare	0.696	0.016
4.	8 weeks	Partner happy with the way mother brings up child	0.503	0.023
5.	8 weeks	Home is woman's place, no part for me	0.649	0.024
6.	8 weeks	Partner always getting under mother's feet	0.573	0.017
7.	8 weeks	Mother dislikes partner being involved with childcare	0.718	0.027
**III**	**Paternal beliefs regarding mother-father relationship and parenting**			
1.	8 weeks	Parenthood has made partner and mother closer	0.363	0.017
2.	8 weeks	Mother no longer gives partner attention	0.709	0.018
3.	8 weeks	Partner feels hurt by attention mother gives child	0.758	0.024
**IV**	**Paternal beliefs regarding employment and parenthood**			
1.	8 weeks	Mother expects partner to take child after work	0.514	0.006
2.	8 weeks	Partner too tired to take child after work	0.898	0.004
3.	8 weeks	Partner takes child after work, so mother is free	0.708	0.006
4.	8 weeks	Partner finds child hard to manage after work	0.899	0.004
5.	2 years 9 months	Partner finds it hard to cope with child after work	0.303	0.016
	**Latent factor model fit indices**			
	Free parameters			118
	Root Mean Square Error of Approximation (RMSEA)			0.044 (95%CI 0.043 to 0.045)
	Comparative Fit Index (CFI)			0.957
	Tucker-Lewis Index (TLI)			0.950

Table S3. *Associations between child-focused and mother-influenced dimensions of paternal involvement*

Paternal involvement factors		Point estimate (β)[a]	S.E.	p-value
**Six specific factors capturing child-focused dimensions of paternal involvement**				
Paternal parenting confidence	Paternal conflictual relationship with child	0.682	0.014	≤0.001
	Paternal enjoyment and warmth	0.687	0.013	≤0.001
	Paternal beliefs regarding caregiving	-0.001	0.025	0.836
	Paternal involvement in childcare	0.241	0.017	≤0.001
	Paternal worries about child	0.294	0.020	≤0.001
Paternal conflictual relationship with child	Paternal enjoyment and warmth	0.695	0.011	≤0.001
	Paternal appreciation of routine	0.057	0.023	0.017
	Paternal involvement in childcare	0.077	0.018	≤0.001
	Paternal worries about child	0.277	0.018	≤0.001
Paternal enjoyment and warmth	Paternal appreciation of regular routine	0.223	0.022	≤0.001
	Paternal involvement in childcare	0.260	0.016	≤0.001
	Paternal worries about child	0.002	0.019	0.904
Paternal beliefs regarding caregiving	Paternal involvement in childcare	0.066	0.024	0.006
	Paternal worries about child	-0.116	0.026	≤0.001
Paternal involvement in childcare	Paternal worries about child	-0.062	0.020	0.002
**Four specific factors capturing mother-influenced dimensions of paternal involvement**				
Paternal perceptions of maternal 'gatekeeping'	Paternal beliefs regarding mother-father relationship and parenting	0.740	0.020	≤0.001
	Paternal help with household tasks	0.209	0.018	≤0.001

	Paternal beliefs regarding employment and parenthood	0.283	0.017	≤0.001
Paternal beliefs regarding mother-father relationship and parenting	Paternal help with household tasks	0.165	0.019	≤0.001
	Paternal attitudes to employment and parenthood	0.302	0.017	≤0.001
Paternal help with household tasks	Paternal beliefs regarding employment and parenthood	-0.002	0.014	0.898

Note: [a] Effect size are standardised (the variance of the latent factors was fixed to ‘1’) regression coefficients (*β*)

Table S4. *Distribution of sociodemographic characteristics in the original Avon Longitudinal Study of Parents and Children (ALSPAC) Cohort and the study complete and imputed samples*

Sample demographic characteristics assessed during pregnancy [1]	Core ALSPAC sample [a] (n=14,901)	Complete sample [b] (n=3,434)	Imputed sample [c] (n=6,029)
	(%)	(%)	(%)
Maternal educational attainment			
A-Level/Degree	62.4	52.0	56.7
Minimal education/none/O-Level	37.6	48.0	43.3
Presence of financial difficulties			
Financial difficulties	78.8	85.4	82.7
No financial difficulties	21.2	14.6	17.3
Homeownership status			
Owned/mortgaged	79.0	89.7	84.3
Private/council rented	21.0	10.3	15.7
Marital status			
Married	75.0	86.9	82.7
Never married	25.0	13.1	17.3
	Mean (SD)	Mean (SD)	Mean (SD)
Parental conflict	10.01 (1.77)	10.27 (1.66)	10.13 (1.70)
Maternal age	27.23 (4.96)	28.73 (4.28)	28.09 (4.59)
Paternal PND	3.35 (3.70)	3.18 (3.50)	3.41 (3.67)

Note: [1] Additional missing data on demographics: maternal educational attainment missing for 3,237/21.7%; maternal age missing for 931/6.0%; financial difficulties missing for 2,292/14.6%; homeownership status missing for 4,699/30.0%; marital status missing for 2,092/13.4%; parental conflict missing for 3,562/22.8%; paternal PND missing for 8,572/54.8%.

[a] Core ALSPAC sample: no exposure or outcome data; [b] Complete sample: exposure, outcomes and confounders data available; [c] Imputed sample: imputed missing data on exposure, outcomes and confounders (except child PGS).

PGS: polygenic score for neuroticism; Paternal PND: paternal postnatal depression.

Table S5. *Estimates of direct and mediated effects in the unadjusted model and models adjusted for child PGS, gender, antenatal baseline confounders, and paternal PND as an intermediate confounder in imputed sample (n=6,029)*

Effect Size [1]	Model estimates (n=6,029) Unadjusted model		Model 1[a]		Model 2[b]	
	B [95% CI]	P-value	*B* [95% CI]	P-value	*B* [95% CI]	P-value
**Child-focused dimensions of paternal involvement**						
1. Total indirect effect[*]	0.098 [0.074, 0.121]	≤0.001	0.083 [0.061, 0.104]	≤0.001	0.054 [-0.030, 0.138]	0.209
2. Direct effect[**]	0.142 [0.110, 0.175]	≤0.001	0.118 [0.085, 0.151]	≤0.001	0.166 [0.088, 0.244]	≤0.001
3. Total effect [***]	0.240 [0.212, 0.267]	≤0.001	0.201 [0.173, 0.228]	≤0.001	0.220 [0.061, 0.379]	0.006
4. Specific indirect effects						
Paternal parenting confidence	0.027 [0.019, 0.035]	≤0.001	0.025 [0.017, 0.033]	≤0.001	0.012 [-0.023, 0.047]	0.491
Paternal conflictual relationship with child	0.042 [0.030, 0.054]	≤0.001	0.038 [0.028, 0.048]	≤0.001	0.042 [0.005, 0.079]	0.028
Paternal enjoyment and warmth	0.014 [0.010, 0.020]	≤0.001	0.012 [0.006, 0.018]	0.001	-0.005 [-0.025, 0.015]	0.625
Paternal involvement in childcare	0.001 [-0.001, 0.003]	0.488	0.001 [-0.001, 0.003]	0.352	0.001 [-0.001, 0.003]	0.602
Paternal beliefs regarding caregiving	0.001 [-0.001, 0.003]	0.634	0.001 [-0.001, 0.003]	0.832	-0.001 [-0.005, 0.003]	0.520
Paternal worries about child	0.014 [0.006, 0.022]	≤0.001	0.008 [0.002, 0.014]	0.005	0.006 [0.001, 0.012]	0.037
**Mother-influenced dimensions of paternal involvement**						
1. Total indirect effect	0.021 [0.005, 0.037]	0.006	0.002 [-0.010, 0.014]	0.716	-0.014 [-0.034, 0.006]	0.193

2. Direct effect**	0.222 [0.193, 0.251]	≤0.001	0.197 [0.166, 0.228]	≤0.001	0.191 [0.162, 0.220]	≤0.001
3. Total effect ***	0.243 [0.215, 0.270]	≤0.001	0.199 [0.171, 0.226]	≤0.001	0.177 [0.086, 0.148]	≤0.001
4. Specific indirect effects						
Paternal help with household tasks	0.001 [-0.001, 0.003]	0.535	0.001 [-0.001, 0.003]	0.784	-0.005 [-0.017, 0.007]	0.397
Paternal perception of maternal 'gatekeeping'	0.015 [0.003, 0.027]	0.007	0.002 [-0.010, 0.014]	0.649	-0.008 [-0.020, 0.004]	0.191
Paternal beliefs regarding mother-father relationship and parenting	0.001 [-0.007, 0.009]	0.687	0.001 [-0.005, 0.007]	0.941	0.001 [-0.001, 0.003]	0.761
Paternal beliefs regarding employment and parenthood	0.005 [0.001, 0.010]	0.006	0.001 [-0.001, 0.003]	0.726	0.001 [-0.001, 0.003]	0.994

Note:[1] Effect size are unadjusted and adjusted regression coefficients (*B* unstandardised); unadjusted model (exposure, outcome and mediators only); Model 1: adjusted for child PGS and antenatal baseline confounders (child gender, financial problems, homeownership status, maternal age and education, parental conflict, marital status); Model 2: further adjusted for paternal PND at 8 months as an intermediate confounder.

Model 1: * Total indirect effect = maternal PND->aspects of paternal engagement->offspring emotional and behavioural development; **Direct effect = maternal PND-> offspring emotional and behavioural development; ***Total effect = total indirect effect + direct effect.

Model 2: * Total indirect effect = maternal PND->aspects of paternal engagement->offspring emotional and behavioural development + maternal PND->paternal PND->aspects of paternal engagement -> offspring emotional and behavioural development; **Direct effect = maternal PND-> offspring emotional and behavioural development + maternal PND->paternal PND->offspring emotional and behavioural development; ***Total effect = total indirect effect + direct effect.

Maternal PND: maternal postnatal depression; PGS: polygenic score for neuroticism; Paternal PND: paternal postnatal depression.
